# Weld Width Measurement and Defect Detection Method for Desiccant Cartridges Based on Machine Vision

**DOI:** 10.3390/s26175499

**Published:** 2026-08-30

**Authors:** Qixuan Wang, Songxiao Cao, Sujuan Xiang, Dong Zhang, Tao Song, Zhipeng Xu, Qing Jiang

**Affiliations:** 1 College of Metrology Measurement and Instrument, China Jiliang University, Hangzhou 310018, China; p24020854104@cjlu.edu.cn (Q.W.); songtao@cjlu.edu.cn (T.S.); xuzhipeng@cjlu.edu.cn (Z.X.); 06a0203051@cjlu.edu.cn (Q.J.); 2 Hangzhou Institute of Quality and Metrology, Hangzhou 310018, China; xiangsujuan@126.com (S.X.); zhangdong_1005@163.com (D.Z.)

**Keywords:** desiccant cartridge, laser transmission welding, machine vision, B-spline dynamic mask, orthogonal projection, defect polarity classification

## Abstract

In industrial automated production, reliable inspection of welded desiccant cartridges is challenging because polypropylene welds exhibit irregular boundaries, low-contrast defects, and non-uniform surface reflections. This study proposes a machine vision-based method for integrated weld width measurement and multi-type weld defect detection. A cubic B-spline fitting strategy is first employed to extract the weld boundaries and suppress interference from non-weld regions. The weld width is then measured along the local normal direction using an orthogonal projection method, with the minimum width used to identify excessively narrow welds and weld breakage. For pore and burn-through detection, distance transform-based watershed segmentation is combined with grayscale polarity analysis to separate true defects from pseudo-defects. The polarity thresholds are determined using an independent calibration batch and further verified on a separate production batch before evaluation on an independent 500-sample test set. Experimental results show that the proposed method achieves an average IoU of 96.7% for weld region extraction. The average and minimum width absolute errors are 0.0509 mm and 0.0438 mm, respectively, with relative errors of 1.69% and 1.72%. Compared with four representative geometric width measurement methods, the proposed method achieves the lowest measurement errors while requiring only 1.84 ms for width measurement. On the independent 500-sample test set, the proposed system achieves an overall defect detection accuracy of 97.20% and a Macro-F1 score of 97.82%. The average end-to-end processing time is 55.48 ms per sample, demonstrating the potential of the proposed method for real-time weld quality inspection of desiccant cartridges under the evaluated industrial imaging conditions.

## 1. Introduction

**Figure 1:**
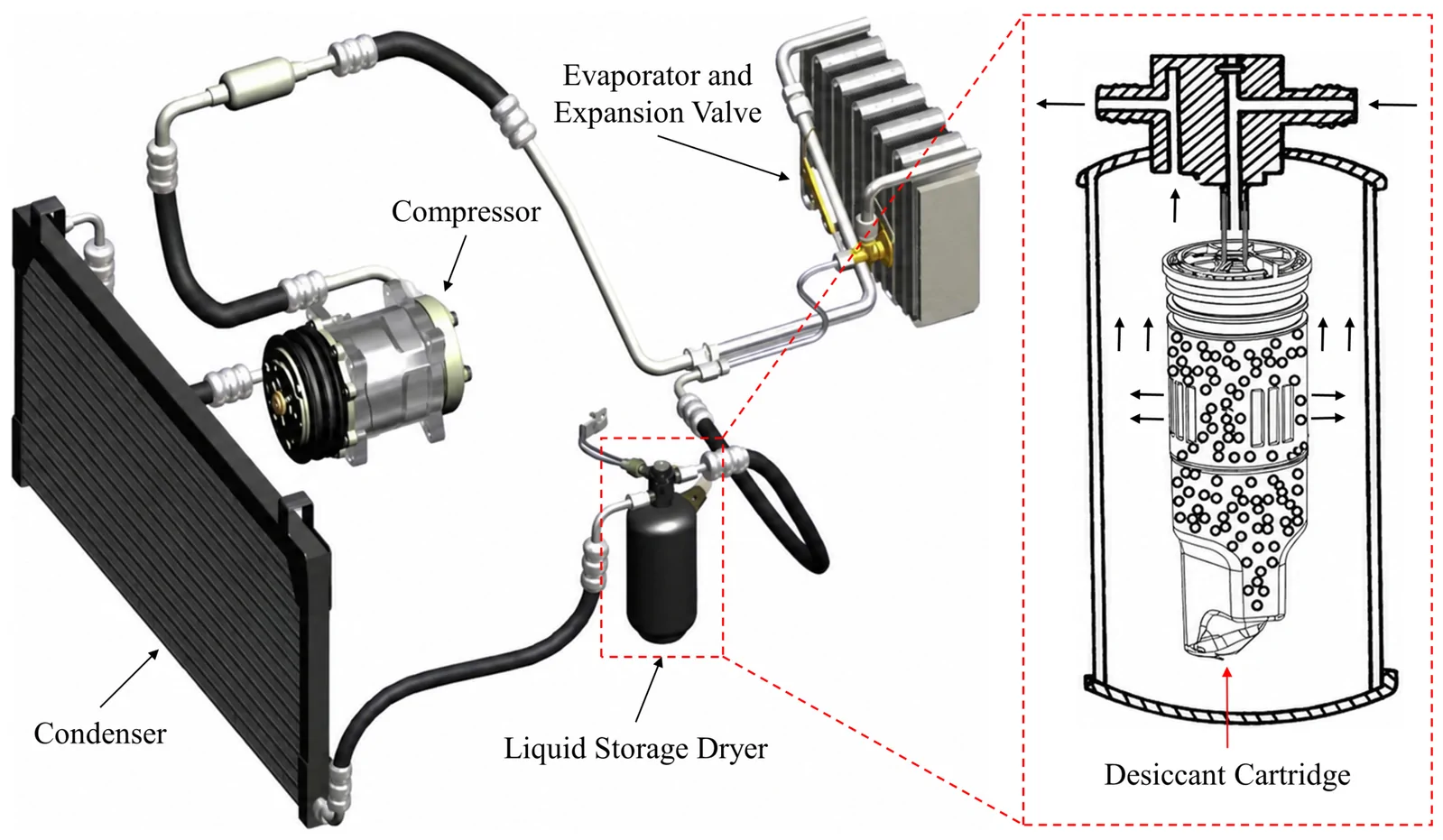
Electric vehicle refrigeration system and its drying and filtering structure.

As the core drying and filtering component of the refrigeration system in electric vehicles, the desiccant cartridge is mainly responsible for removing trace moisture and impurities mixed in the refrigerant fluid, effectively preventing damage to the compressor and other components, as shown in Figure 1. To ensure the sealing performance of the desiccant cartridge, laser transmission welding technology is usually adopted during the assembly process. However, due to external disturbances, such as differences in crystallinity distribution of polypropylene materials, mechanical vibration, and fluctuations in laser energy density, defects such as pore, burn-through, incomplete welding, and excessively narrow width may occur in the weld seam [1]. These defects will damage the sealing integrity of the component, thereby affecting the working reliability of the entire thermal management system. Therefore, weld seam defect detection is crucial for ensuring the overall performance and service life of the thermal management system in electric vehicles.

In this study, the inspection scope is specifically defined according to the defect characteristics observed in the target desiccant cartridge production process and the information available from the adopted 2D vision configuration. Four representative defect categories, namely pore defects, burn-through defects, weld width abnormalities, and weld fractures, are considered. These defects can be effectively characterized through the geometric continuity, weld width, local grayscale distribution, and grayscale polarity of the imaged weld region. Other possible welding defects, such as inclusions, weld displacement, and cracks, are outside the current experimental scope because their reliable characterization may require additional geometric information, higher-resolution imaging, or complementary sensing modalities. Therefore, the objective of this study is not to establish a universal inspection method for all welding defects, but to develop a fast and interpretable vision-based inspection strategy for the major defect modes that can be reliably observed in the target polypropylene laser welding process.

**Figure 2:**
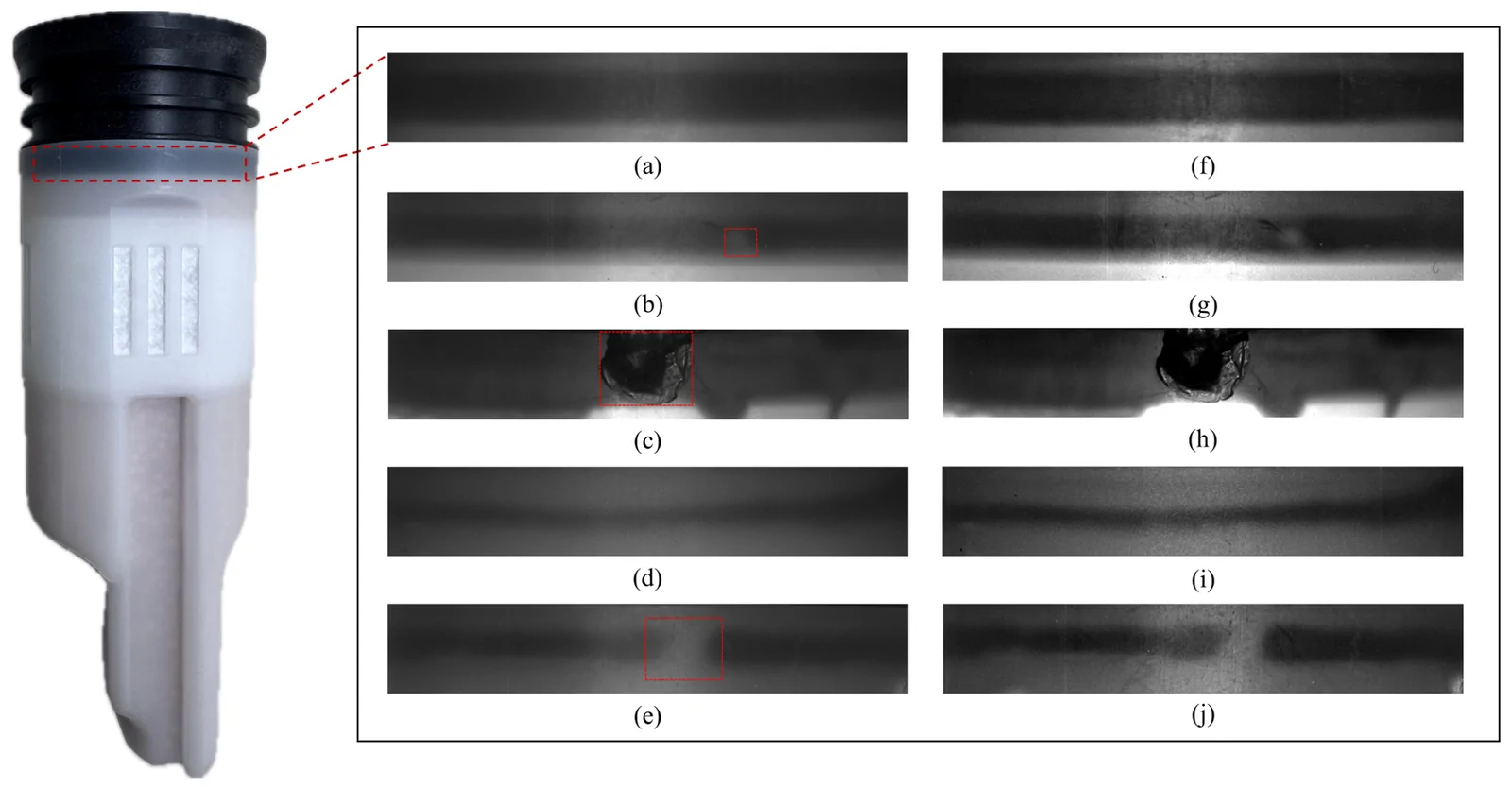
Physical diagram of desiccant cartridge and different types of defects. Defects are marked with red rectangles: (**a**) normal weld; (**b**) pore defect; (**c**) burn-through defect; (**d**) excessively narrow width; (**e**) weld breakage defect; (**f**) normal weld after image enhancement; (**g**) pore defect after image enhancement; (**h**) burn-through defect after image enhancement; (**i**) excessively narrow width after image enhancement; (**j**) weld breakage defect after image enhancement.

Figure 2 shows the morphological characteristics of these four types of weld defects observed in the weld: (a) is a defect-free weld; (b) is a pore defect, which is characterized by the presence of white cavities in the weld; (c) is a burn-through defect, which is characterized by the presence of dark patches in the weld; (d) is an excessively narrow weld, which is characterized by a weld width smaller than the specified value; (e) is a weld breakage defect, which is characterized by obvious fractures in the weld area; (f–j) are images of the normal weld and the above four defects after image enhancement.

For the quality inspection of laser transmission welding, existing systems and processes are mainly divided into two categories: destructive testing and non-destructive testing [2]. Traditional destructive testing methods such as tensile testing, bending testing, and impact toughness testing can accurately evaluate the strength, ductility, and impact resistance of welds, but they are time-consuming, costly, and cannot be applied to online full inspection [3,4]. In terms of non-destructive testing, although ultrasonic testing and X-ray testing technologies are relatively mature, they often present difficulties in meeting the beat requirements of large-scale production lines due to expensive equipment and low detection efficiency [5]. In recent years, optical coherence tomography and infrared thermal imaging technology have gradually attracted attention due to their high-precision characteristics. For example, Jiang et al. [6] established an online detection system based on optical coherence tomography technology to measure the pore defects of aluminum alloy laser-welded workpieces in real time, with a detection accuracy of up to 20 μm. Venkatraman et al. [7] verified the heat transfer model by applying in situ infrared thermal imaging technology and established an energy evaluation framework that correlates process parameters with weld interface shear strength, realizing the quantitative prediction of weld quality in ultrasonic additive manufacturing. However, the optical coherence tomography system has a complex optical structure, is sensitive to vibration, and has extremely high maintenance costs; infrared thermal imaging is easily interfered with by ambient temperature and has a long data processing time, making it difficult to meet the requirements of high-tempo production in industrial sites [8]. In contrast, machine vision-based detection technology has become the most potential online detection solution in industrial sites due to its advantages of non-contact, high speed, low cost and strong system flexibility [9]. Nevertheless, most current machine vision weld defect detection systems are mainly aimed at metal welds with high reflectivity and high contrast, while research on the special optical properties of translucent polypropylene materials is relatively scarce. Prabhakaran et al. [10] pointed out that translucent materials have light scattering and transmission phenomena, resulting in indistinct grayscale differences and blurred boundaries between the weld area and non-weld area during imaging, which makes the existing general visual inspection systems often have false or missed detections when facing desiccant cartridge weld inspection, and it is difficult to meet the high-precision measurement requirements. At the same time, it also puts forward higher challenges for the light source design and image acquisition of the imaging system.

At the level of image processing algorithms, there are currently two main research directions: intelligent algorithms based on deep learning and traditional algorithms based on digital image processing. With the development of artificial intelligence, deep learning has shown strong performance in the field of industrial defect detection. A review by Zhang et al. [11] pointed out that models such as CNN, YOLO, and U-Net have significantly higher accuracy than traditional methods in handling weld defect detection tasks with complex backgrounds and variable defect morphologies. Zhu et al. [12] proposed a welding defect recognition method based on a CNN architecture, which achieved an accuracy of 98.75% on a dedicated database by extracting high-level features of weld surface defect images and combining them with a random forest classifier. Li et al. [13] further improved YOLOv8s by introducing RepGFPN, small target detection heads, FocalNets, and the CGA attention mechanism, effectively enhancing the detection accuracy and generalization ability of welding defects in aluminum liquid storage tanks. Zhang et al. [14] first applied 3D U-Net to phased array ultrasonic testing data of ship welds and, by constructing a dedicated dataset and optimizing skip connections and residual modules, achieved a Dice coefficient of 90.9% with a lightweight model, providing a feasible solution for automatic segmentation of complex defects. Seo et al. [15] adopted a transfer learning framework of ResNet and AlexNet to build a classification model for gas metal arc welding weld appearance defects and verified the effectiveness of stochastic gradient descent and mini-batch training through hyperparameter optimization.

Although deep learning has shown great potential in the field of visual detection, its specific implementation in the industrial site of desiccant cartridges still faces severe challenges. On the one hand, deep learning models are mainly good at qualitative classification of defects but have inherent limitations in size measurement tasks. The output of neural networks is mostly probability maps or bounding boxes, which makes it difficult to directly provide accurate geometric dimensions. Especially for low-contrast translucent polypropylene materials, the regression accuracy of the model for edge positions is limited, making it difficult to meet the measurement accuracy required by the process [16,17,18]. On the other hand, the inherent black-box nature of neural networks makes their decision-making process lack interpretability [19]. Once a false detection or missed detection occurs, on-site engineers have difficulty tracing and locating the root cause. This uncertainty often becomes a key bottleneck restricting its practical application in the automotive supply chain with a strict quality traceability system. In contrast, traditional image processing algorithms based on edge detection, threshold segmentation, and morphological operations, with their characteristics of low computational complexity, fast running speed, algorithmic transparency, and ease of deployment, demonstrate better comprehensive applicability in industrial scenarios with high real-time requirements and limited samples. Jin et al. [20] proposed a weld width measurement method based on machine vision, which realizes weld region segmentation through a dedicated hardware platform and the GrabCut algorithm, achieving a measurement accuracy of 0.1 mm and a detection speed of 30.7 mm/s, fully verifying the effectiveness of traditional geometric methods in precision measurement. Guo et al. [21] designed a weld joint detection system that integrates Otsu threshold segmentation, morphological processing, and a random incremental algorithm to accurately extract contours, and then uses the watershed algorithm and difference operation to achieve accurate segmentation of adhered weld seams and rapid localization of missing defects, meeting the real-time quality inspection requirements. Sun et al. [22] developed an online detection and classification algorithm combining an improved Gaussian mixture model with machine vision for welding defects in thin-walled metal cans. After optimizing the classifier parameters on the real production line, the online detection accuracy exceeded 99%, which can stably support continuous real-time detection. Gao et al. [23] addressed the tracking challenge of variable gap fillet welds under strong reflection and noise interference, proposing a position detection and feature extraction method based on the column gray difference operator and optimized RANSAC algorithm. Under high-speed welding conditions, the average detection error is less than 0.23 mm, showing excellent anti-interference ability.

Although traditional image processing algorithms have advantages in real-time performance and interpretability, they still have obvious limitations when dealing with desiccant cartridge made of translucent polypropylene material: affected by the light scattering characteristics of the material and the non-uniform illumination of the cylinder, the image contrast is extremely low and the edges are blurred, resulting in problems such as incomplete segmentation, blurred edges and the introduction of a large number of pseudo-defects in traditional global thresholding or conventional edge detection, and it is difficult to achieve accurate quantification of the width of complex curved welds. To address the above problems, this paper adopts a specifically designed traditional image processing algorithm combined with dynamic geometric masks and multi-dimensional feature mapping to achieve high-precision online detection under low-contrast conditions.

Accordingly, the main contributions of this study are summarized as follows:

1. Dynamic geometry-constrained weld localization for translucent polypropylene. To address the low contrast, blurred boundaries, and background interference of translucent polypropylene laser-welded joints, a dynamic ROI construction strategy based on cubic B-spline fitting is developed. Instead of using a fixed detection region, the proposed strategy adaptively follows the weld boundary and constrains subsequent processing to the actual weld region, thereby improving the robustness of weld localization against non-weld background interference.
2. Geometry-consistent weld width measurement for curved weld joints. To address the geometric deviation caused by measuring non-straight welds along fixed image coordinate directions, a local normal-based orthogonal projection strategy is developed to measure the weld width along the local geometry of the weld boundary. The resulting width measurements are further used to identify excessively narrow welds and weld breakage, thereby integrating quantitative dimensional inspection with weld defect classification.
3. Grayscale polarity-aware discrimination of pore and burn-through defects. To address the difficulty of distinguishing true defects from pseudo-defects caused by reflections and low-contrast regions, a marker-controlled watershed strategy based on Euclidean distance transformation is combined with local grayscale polarity verification. The watershed step separates connected defect candidates, while the polarity feature evaluates the relative grayscale characteristics between each candidate defect and its local background, enabling the discrimination of pores and burn-through defects and the suppression of reflection-induced pseudo-defects.

The structure of this paper is as follows: Section 2 describes the configuration of the detection system, Section 3 introduces the detection method, including image preprocessing, weld segmentation and defect identification. Section 4 outlines the relevant experiments, and Section 5 provides the corresponding conclusions.

## 2. Vision System Design

**Figure 3:**
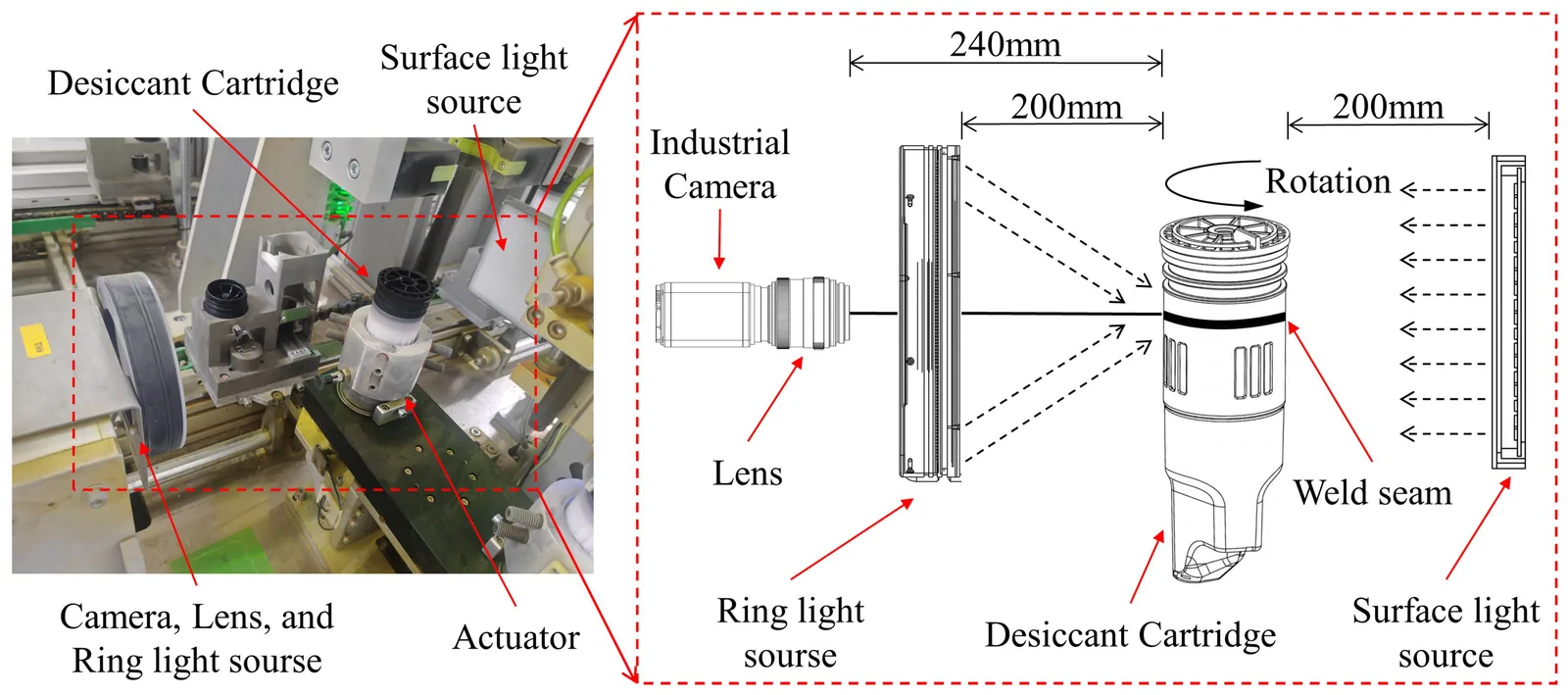
Imaging schematic diagram of weld defect detection system for desiccant cartridge.

This study focuses on acquiring high-quality two-dimensional images of desiccant cartridge welds during the production process. The polypropylene desiccant cartridges investigated in this study were welded using a HWD100-SM laser welding system (Han’s Laser, China) set to laser transmission welding mode. The laser power, welding time, and focal length were set to 30 W, 3 s, and 100 mm, respectively. These parameters were kept constant during sample preparation to ensure consistent welding conditions for all experimental samples. The resulting welded joints were subsequently inspected using the proposed machine vision system. The detection system and imaging method are shown in Figure 3. The system drives the desiccant cartridge to rotate at a constant speed through a servo motor, and the industrial camera triggers image acquisition during the rotation process, thereby completely covering the weld area.

Specifically, this system adopts the Hikvision MV-CS016-10GM industrial area scan camera, produced by Hikvison, a company based in Hangzhou, China, as the image acquisition device. The camera has a resolution of 1440 × 1080, a pixel size of 3.45 μm, supports global shutter, and has a maximum frame rate of 65.2 fps, which can effectively eliminate motion blur in rotational motion and perfectly meet the dynamic imaging requirements of desiccant cartridge with constant-speed rotation of servo motors. The camera is equipped with an MVL-MF1628M-8MP FA lens and is installed 240 mm directly in front of the product surface. The camera’s field of view is 87 mm × 65 mm, with a pixel accuracy of 0.06 mm/pix, which can completely cover the welding area of the desiccant cartridge in a single frame image. The light source uses MV-LRDS-120-30W ring white light as front illumination, combined with an MV-LBSS-100-100W surface light source for backlight transmission imaging, which effectively enhances the contrast of defect features in the weld area and reduces the reflection interference of translucent polypropylene material.

## 3. Methods

### 3.1. Overview

**Figure 4:**
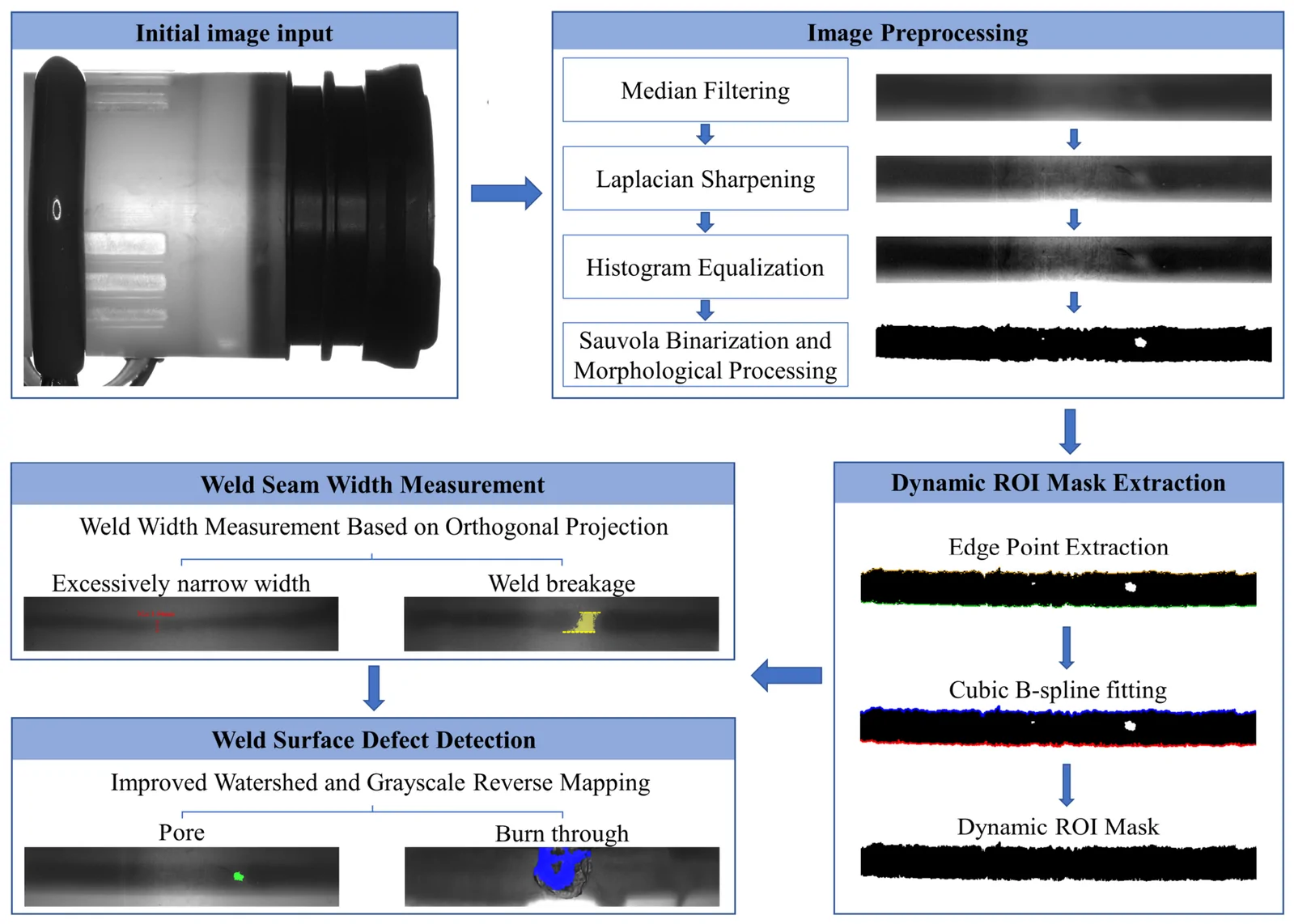
Algorithm flow for measurement of weld seam width and defect detection in desiccant cartridge.

The overall framework of the weld width measurement and defect detection algorithm for desiccant cartridge consists of four sequential and interdependent functional modules: image preprocessing, weld mask extraction, weld width measurement, and weld defect detection, as shown in Figure 4. These modules are designed to address distinct sub-tasks that depend on one another; the output of each preceding stage serves as the necessary input constraint for the subsequent stage.

1. Image preprocessing: The input image first undergoes median filtering, Laplacian sharpening, histogram equalization, Sauvola adaptive binarization, and morphological operations. This stage aims to enhance the grayscale contrast between the weld region and the background under the low-contrast and light-scattering conditions of translucent polypropylene, thereby providing a reliable binary foundation for subsequent geometric extraction.
2. Weld mask extraction: Building upon the preprocessed binary image, cubic B-spline fitting is applied to the upper and lower weld edges to construct a dynamic ROI mask. This mask effectively suppresses interference from the translucent polypropylene cylinder and the black end-cap background and provides a precise geometric calculation domain that is indispensable for both accurate width measurement and defect localization.
3. Weld width measurement: Within the region constrained by the dynamic mask, the orthogonal projection algorithm computes the average width and minimum width along the local normal direction of the fitted edge curves. The resulting width metrics are then used to identify two types of geometric defects, excessively narrow width and weld breakage, according to the process specifications.
4. Weld defect detection: Still under the strict spatial constraint of the same dynamic mask, Euclidean distance transform combined with marker-controlled watershed segmentation is first applied to separate candidate defect connected domains. Subsequently, a grayscale inverse-mapping polarity verification mechanism is employed to classify pores and burn-through defects and to filter out pseudo-defects. Because the polarity analysis operates only on the regions already isolated by the watershed stage and confined by the dynamic mask, this final classification step is directly dependent on the preceding modules.

In summary, the four modules form a sequential processing pipeline in which each stage relies on the geometric or binary constraints established by the previous stages. Removing or replacing any individual module would interrupt the corresponding downstream task rather than merely degrade an otherwise identical system, which is why the present method is evaluated as an integrated inspection framework.

### 3.2. Dynamic ROI Mask Extraction Based on Cubic B-Spline Fitting

After image preprocessing, the main binary connected domain of the weld has been basically formed. However, due to the mechanical vibration of the desiccant cartridge and the fluctuation of laser energy density, the physical boundary of the weld is often not an ideal horizontal straight line, but a smooth curve with a slight curvature. If rigid ROI is still used for interception, it will inevitably lead to the inclusion of both the binarized white cylinder and the heterogeneous background of the black end cap in the ROI, which seriously affects the stability of subsequent width measurement and defect detection. In order to completely strip the background interference of the translucent polypropylene cylinder and the black end cap and provide an extremely accurate calculation area for subsequent width measurement and defect detection, this paper introduces cubic B-spline fitting to construct a dynamic ROI mask based on the binary image.

#### 3.2.1. Extraction of Discrete Point Set at Weld Edge

**Figure 5:**
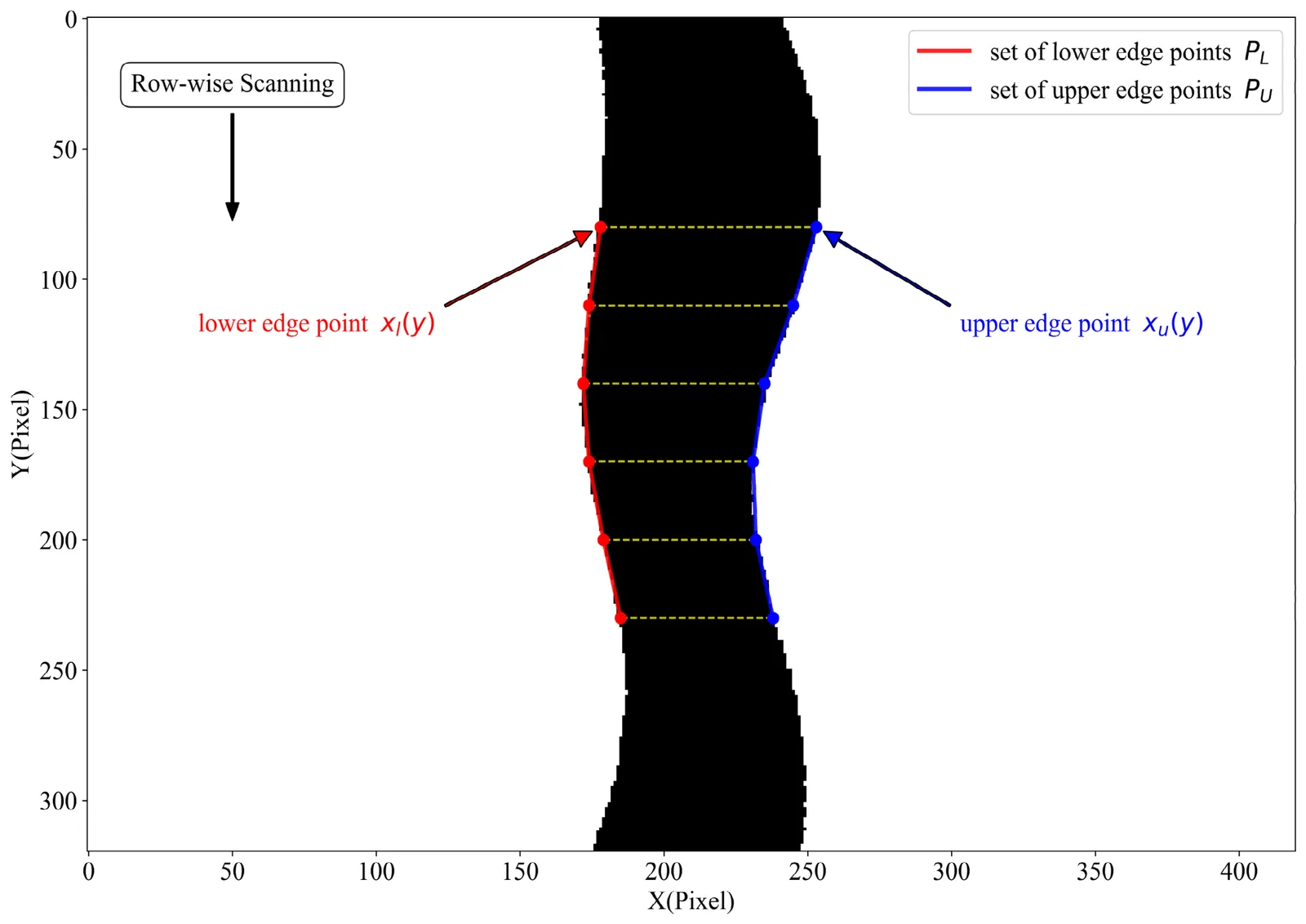
Schematic diagram of extraction principle for discrete point set on weld edge.

As shown in Figure 5, a local image coordinate system is established, and a row-by-row scan is performed on the binary image after image preprocessing. Within the effective weld area of the image, the gray level jump points of the target connected domain are detected along the *x*-axis direction. The first foreground pixel with a gray value of 0 encountered in the *y*-th column is set as the lower edge point, and its abscissa is recorded as *x*_u_(*y*); the last encountered foreground pixel is set as the upper edge point, and its abscissa is recorded as *x*_l_(*y*).

**Figure 6:**
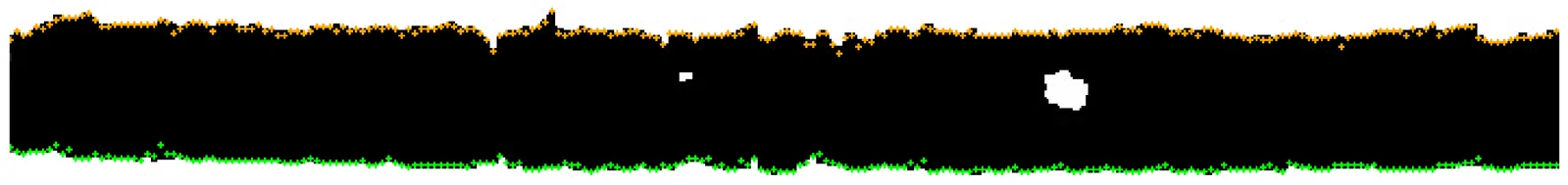
Extracted discrete edge point set of weld seam.

As shown in Figure 6, by traversing all valid rows of the entire image, two original discrete edge point sets, *P*_U_ and *P*_L_, which describe the upper and lower physical boundaries of the weld seam, are finally obtained.

(1) $$P_{U}=\left\{\left(x_{u}(y),y)|y\in [y_{\mathrm{start}},y_{\mathrm{end}}\right]\right\}$$

(2) $$P_{L}=\left\{\left(x_{l}(y),y)|y\in [y_{\mathrm{start}},y_{\mathrm{end}}\right]\right\}$$

wherein [*y*_start_, *y*_end_] represents the pixel distribution interval of the entire weld area on the vertical axis of the image.

#### 3.2.2. Cubic B-Spline Curve Smoothing Fitting and Dynamic ROI Mask Construction

Although the discrete edge point sets *P*_U_ and *P*_L_ extracted row by row basically reflect the geometric contour of the weld, they are still inevitably affected by pixel quantization errors, slight local illumination fluctuations, and weak noise during the scanning process, resulting in slight discontinuities and irregularities of edge points at the sub-pixel level. If subsequent weld width calculation is directly performed based on discrete points, it is easy to introduce measurement errors caused by local fluctuations in the point sets. For this reason, this paper adopts the cubic B-spline interpolation algorithm to smoothly fit these two discrete point sets.

Cubic B-spline curves possess excellent local control and second-order derivative continuity, which can effectively smooth high-frequency noise while perfectly preserving the low-frequency surface geometric deformation features. For a given set of discrete points, the cubic B-spline basis function *N_i_*_,3_(*t*) is defined by the De Boor–Cox recurrence formula:

(3) $$N_{i,0}(t)=\left\{\begin{array}{ll} 1, & t_{i}\leq t<t_{i+1}\\ 0, & \mathrm{otherwise} \end{array}\right.$$

(4) $$N_{i,k}(t)=\frac{t-t_{i}}{t_{i+k}-t_{i}}N_{i,k-1}(t)+\frac{t_{i+k+1}-t}{t_{i+k+1}-t_{i+1}}N_{i+1,k-1}(t),\;(k=1,2,3)$$

A fixed smoothing factor of *s* = 3.0 was adopted to control the trade-off between fidelity to the extracted boundary points and curve smoothness. Specifically, the spline fitting minimizes the deviation between the original boundary points and the fitted curve subject to the prescribed smoothing condition. The knot sequence and spline coefficients are determined automatically by the underlying FITPACK algorithm according to the specified smoothing factor. After fitting, the continuous spline curve is evaluated at 1000 uniformly spaced parameter values over the complete parameter interval of the original boundary data, producing a dense and smooth representation of the weld contour for subsequent ROI construction.

**Figure 7:**
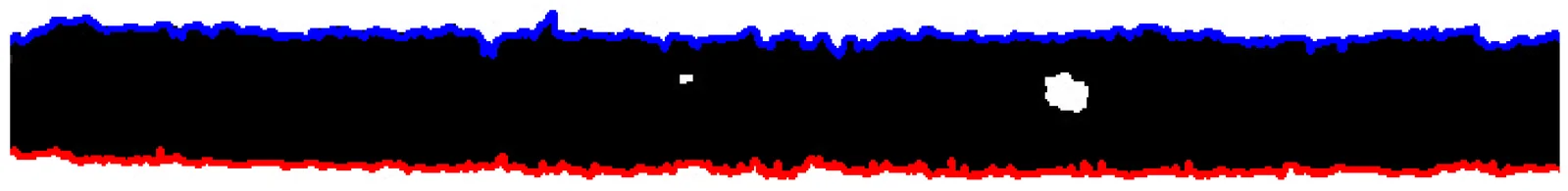
Fitted upper and lower edge curves of the weld.

As shown in Figure 7, after parameterizing the discrete point set and solving the linear equations with boundary conditions to determine the control vertices, the analytical curve functions *C*_upper_(*t*) and *C*_lower_(*t*) that describe the continuous geometric features of the upper and lower edges of the weld can be obtained.

Based on the fitted geometrically continuous boundary curves *C*_upper_(*t*) and *C*_lower_(*t*), the algorithm constructs a dynamic ROI mask *M*_roi_(*x*, *y*) in the 2D image space. For any pixel coordinate (*x*, *y*) in the image, its mask value is defined as

(5) $$M_{\mathrm{roi}}(x,y)=\left\{\begin{array}{ll} 0, & C_{\mathrm{upper}}(y)+\delta \leq x\leq C_{\mathrm{lower}}(y)-\delta \\ 1, & \mathrm{otherwise} \end{array}\right.$$

wherein *C*_upper_(*t*) and *C*_lower_(*t*) represent the calculated values of the upper and lower edge abscissas obtained by cubic B-spline fitting at the ordinate *y*; *δ* is a preset safe shrinkage margin set to 2 pixels, which is used to shield the transition blur zone and artifact interference caused by light scattering at the edge of the translucent polypropylene material.

**Figure 8:**
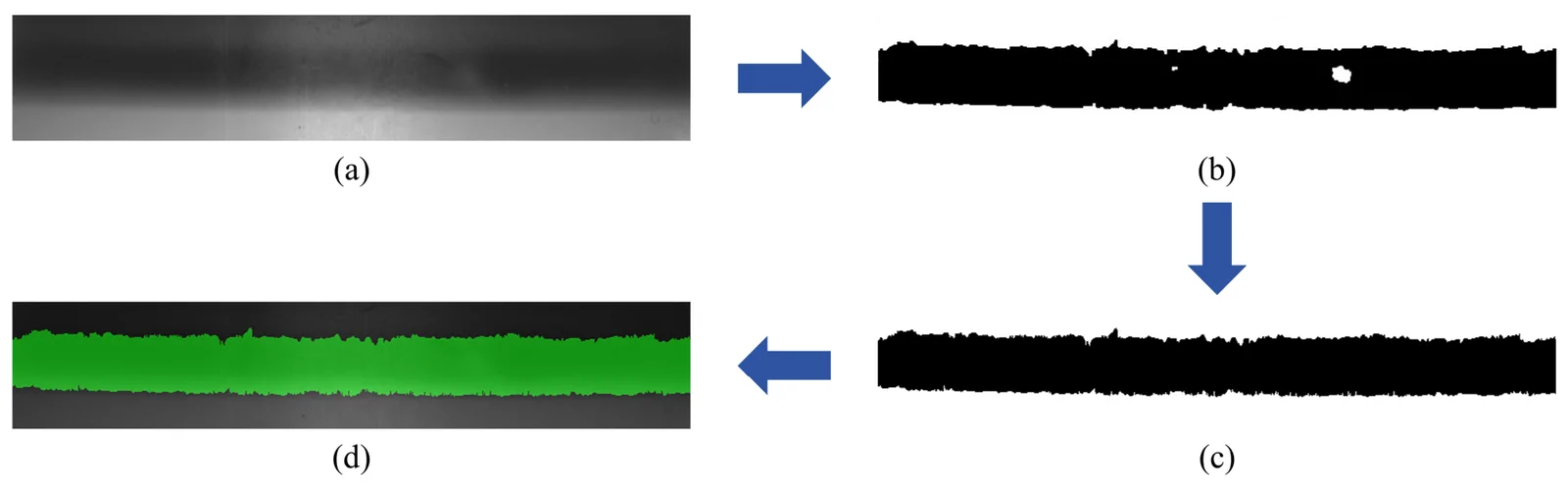
Construction process of dynamic ROI mask. (**a**) Original weld image. (**b**) Preprocessed weld image. (**c**) Dynamic ROI mask. (**d**) Mask overlay effect (green area is the effective weld calculation area).

As shown in Figure 8, the dynamic mask *M*_roi_(*x*, *y*) effectively suppresses the background noise in non-detection areas. The constructed pure mask will serve as the core geometric constraint benchmark for all subsequent weld width measurement and internal defect feature extraction, fundamentally preventing false detection and missed detection caused by uneven background illumination and diffuse reflection.

### 3.3. Weld Width Measurement Algorithm Based on Orthogonal Projection

In traditional machine vision weld width detection, the method of directly calculating the pixel difference between the upper and lower boundaries along the direction of the image coordinate axis is usually used to obtain the target width. However, the tangent direction of the weld edge of the desiccant cartridge often forms a certain angle with the coordinate axis of the image. If the vertical distance is directly used as the weld width, the measurement result will be larger due to the geometric projection effect, which cannot objectively reflect the real physical width of the weld. In the definition of differential geometry, the effective width of the weld should be the cross-sectional width strictly perpendicular to the weld direction. Based on the high-precision weld edge curves *C*_upper_(*t*) and *C*_lower_(*t*) output by cubic B-spline fitting, this paper solves the orthogonal width by calculating the shortest Euclidean distance from the point to the curve.

**Figure 9:**
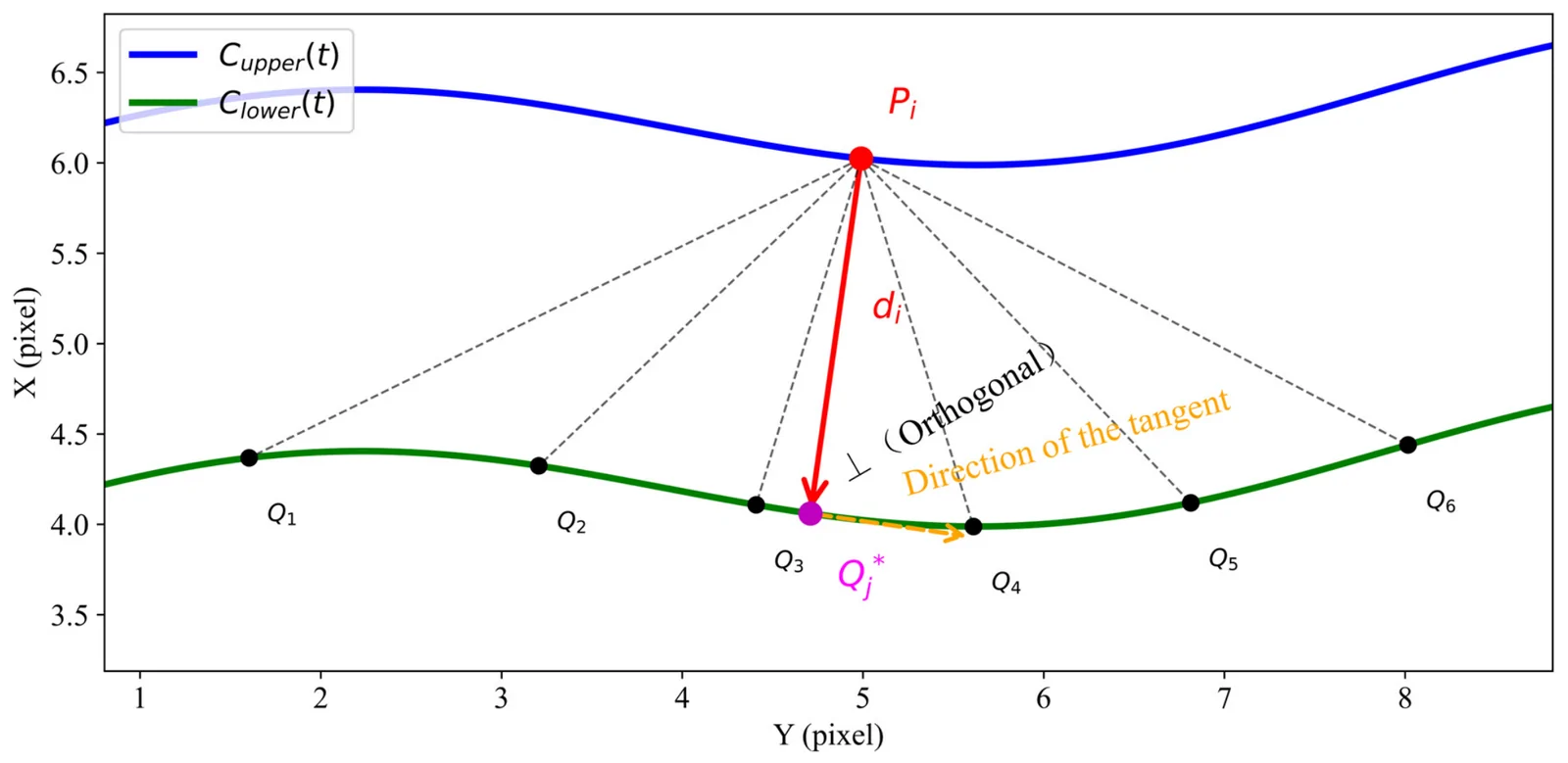
Geometric vector schematic diagram for width calculation based on orthogonal projection.

As shown in Figure 9, for any high-density discrete sampling point *P_i_*(*x_i_*, *y_i_*) on the upper edge curve *C*_upper_, its normal direction represents the true weld width direction at that position. The algorithm searches for a target point $Q_{j}^{*}$ within the corresponding local domain of the lower edge curve *C*_lower_ such that the modulus of the vector *P_i_* $Q_{j}^{*}$ is minimized. At this time, *P_i_* $Q_{j}^{*}$ is the orthogonal projection vector of point *P_i_* on the lower edge, and its length *d_i_* is defined as the instantaneous true physical width at that position. The mathematical model of this process can be expressed as

(6) $$d_{i}=\underset{Q_{j}\in C_{\mathrm{lower}}}{\min}{\left||P_{i}-Q_{j}|\right|}_{2}=\underset{Q_{j}\in C_{\mathrm{lower}}}{\min}\sqrt{{\left(x_{P_{i}}-x_{Q_{j}}\right)}^{2}+{\left(y_{P_{i}}-y_{Q_{j}}\right)}^{2}}$$

where *P_i_* is the *i*-th sampling point on the upper edge fitting curve; *Q_j_* is the search point on the lower edge fitting curve; ||·||_2_ represents the Euclidean norm in 2D space.

To comprehensively assess the welding quality of the desiccant cartridge, the algorithm performs equidistant and high-density sampling along the full length of the weld seam. With a total of *N* sampling points, this process yields a width sequence set that fully characterizes the entire weld area. *D* = {*d*_1_, *d*_2_, …, *d_N_*}. Based on this width sequence, the system calculates and outputs two key quality evaluation indicators: average orthogonal $\bar{W}$ and minimum orthogonal width *W*_min_.

(7) $$\bar{W}=\frac{1}{N}\sum_{i=1}^{N}d_{i}$$

(8) $$W_{\min}=\underset{i}{\min}\left\{d_{i}\right\}$$

Among them, the average $\bar{W}$ is used to evaluate the stability of welding process parameters, while the minimum width *W*_min_ is a critical indicator for identifying weld breakage defects. According to the product quality acceptance specifications used in the actual desiccant cartridge production line, the acceptable weld width should satisfy $\bar{W}$ ≥ 3.5 mm and *W*_min_ ≥ 3.0 mm. These thresholds are predefined industrial acceptance criteria rather than parameters optimized by the proposed algorithm. Due to industrial confidentiality, the detailed specification document cannot be publicly disclosed. After converting pixel measurements into physical dimensions using camera calibration parameters, welds with $\bar{W}$ or *W*_min_ below the corresponding limits are classified as excessively narrow weld defects, while *W*_min_ = 0 indicates a complete weld breakage defect.

Compared with traditional weld width measurement methods, this orthogonal projection model strictly follows the principle of geometric projection and can adaptively handle weld morphologies with arbitrary curvatures, ensuring high precision and high reliability in width detection on the complex geometric surfaces of desiccant cartridge.

### 3.4. Defect Recognition Algorithm Based on Improved Watershed and Grayscale Reverse Mapping

**Figure 10:**
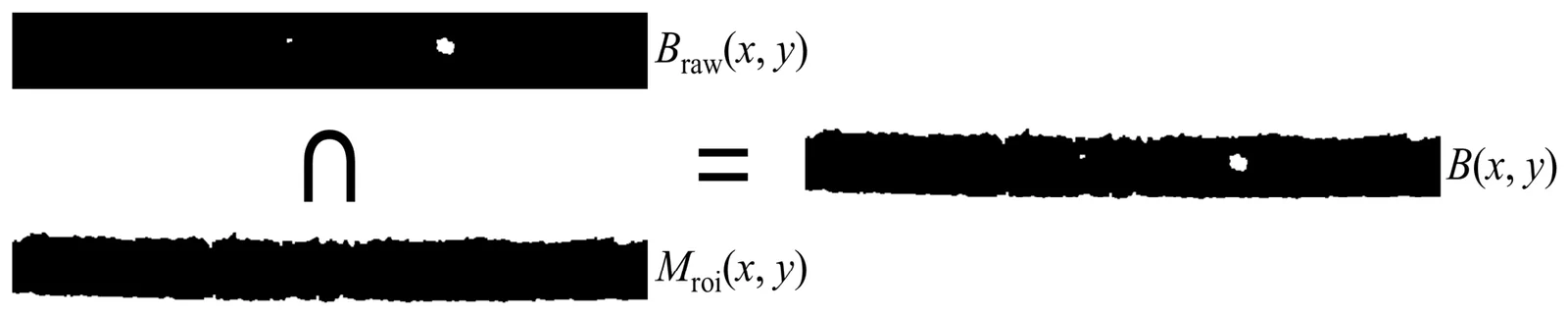
Binary weld defect map after mask constraint.

In the actual detection of desiccant cartridge welds, both pore and burn-through defects exhibit high-frequency mutation characteristics that disrupt the continuity of the weld. To effectively suppress interference from non-detection areas, this paper reuses the weld dynamic mask *M*_roi_(*x*, *y*) established above. The preprocessed binary image *B*_raw_(*x*, *y*) of the foreground defect area is logically ANDed with *M*_roi_(*x*, *y*) to construct a pure defect image to be inspected within the weld area *B*(*x*, *y*), ensuring that all potential defect pixels entering the subsequent detection process are strictly located inside the actual weld, as shown in Figure 10.

(9) $$B(x,y)=B_{\mathrm{raw}}(x,y)\cap M_{\mathrm{roi}}(x,y)$$

On this basis, this paper proposes a defect identification algorithm based on Euclidean distance transform controlled watershed segmentation and grayscale inverse mapping of the original image.

#### 3.4.1. Defect Connected Domain Extraction Based on Euclidean Distance Transform and Marker-Controlled Watershed

In the welds of desiccant cartridge, defects such as pore and burn-through often appear in clusters, and due to complex diffuse reflection and the optical transition effect at the material edges, the binary connected domains of multiple adjacent defects undergo severe physical adhesion in space. If the interwoven connected domains are directly subjected to feature statistics, it will be impossible to accurately determine the actual number of defects and the geometric shape of individual defects. To this end, this paper proposes a connected domain extraction algorithm based on the combination of Euclidean distance transform and marker-controlled watershed.

**Figure 11:**
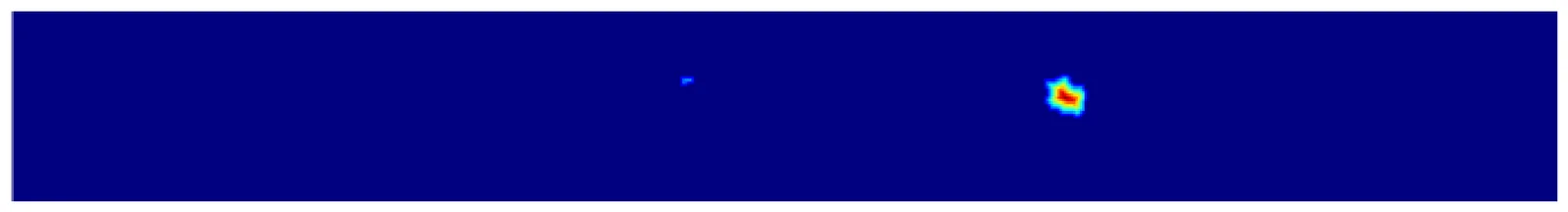
Distance transform map *D*(*x*, *y*).

Let Ω_*f*_ = {(*x*, *y*)∣*B*(*x*, *y*) = 1} be the set of foreground pixels in the potential defect region and Ω_*b*_ = {(*x*, *y*)∣*B*(*x*, *y*) = 0} be the set of background pixels in the normal weld region. For any foreground pixel (*x*, *y*) ∈ Ω_*f*_, calculate the shortest Euclidean distance to the nearest background pixel and construct the distance transform map *D*(*x*, *y*) as shown in Figure 11:

(10) $$D\left(x,y\right)=\underset{\left(x,y\right)\in \Omega_{f}}{\min}\sqrt{{\left(x-x_{b}\right)}^{2}+{\left(y-y_{b}\right)}^{2}}$$

**Figure 12:**
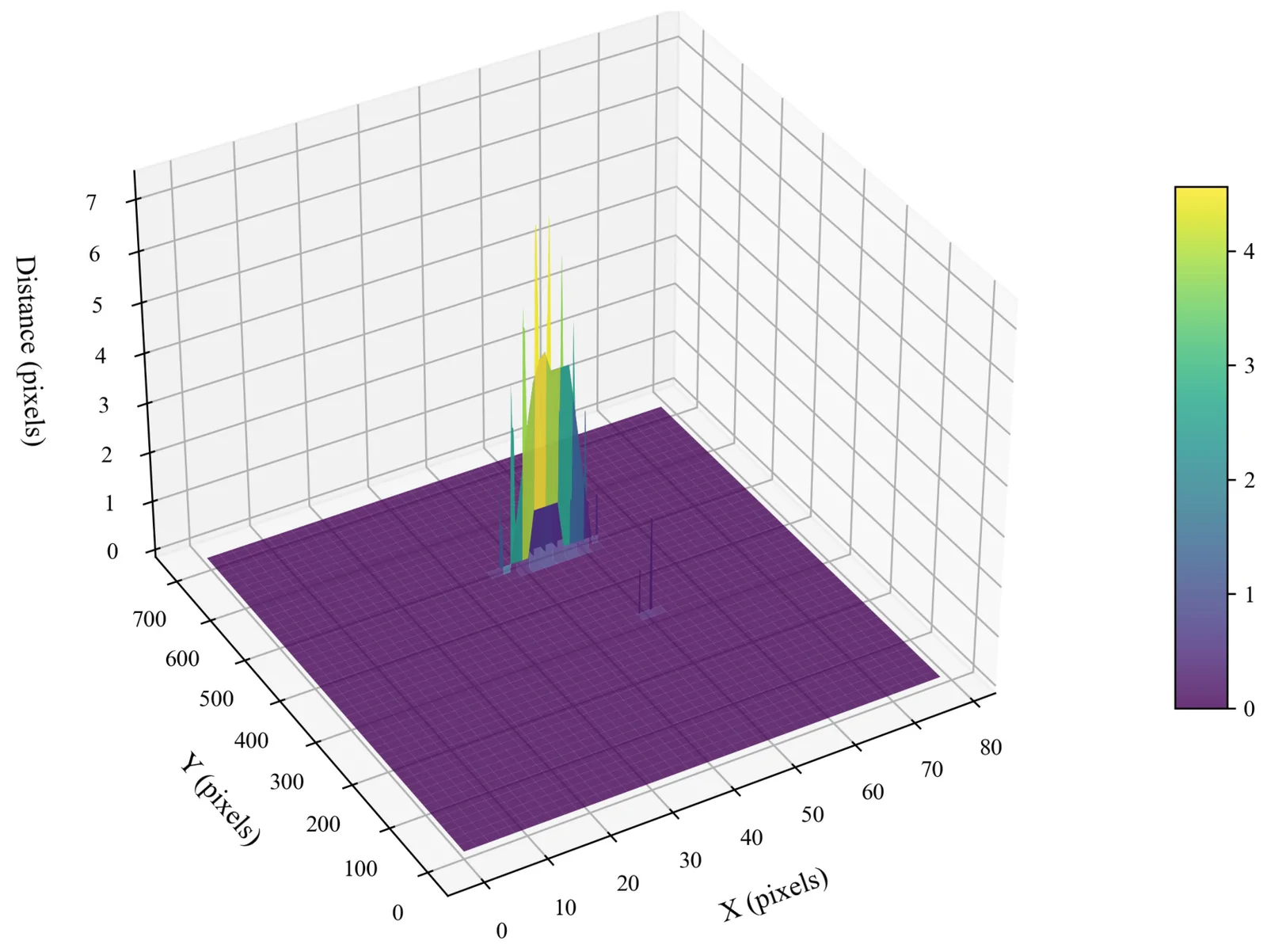
*D*(*x*, *y*) Three-dimensional topological graph.

As shown in Figure 12, in terms of geometric structure, *D*(*x*, *y*) maps the binary connected domain of the plane into a continuous “distance mountain range” with topological height. The numerical distribution characteristics show that the closer to the geometric center of the defect, the larger its value; the closer to the defect boundary, the smaller its value. The local maximum points of the peaks accurately correspond to the geometric centers of each independent defect, while the junctions of defects form relatively low valleys, thereby achieving the preliminary decoupling of adhered defect in the spatial topological structure.

**Figure 13:**
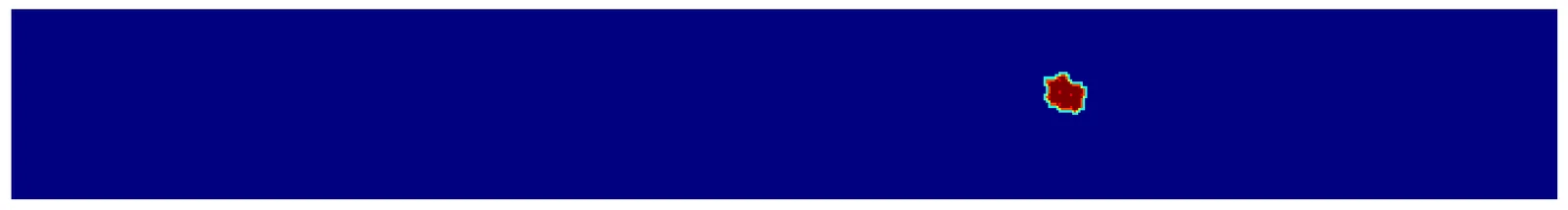
Filtered distance image after morphological reconstruction *D*_f_(*x*, *y*).

**Figure 14:**
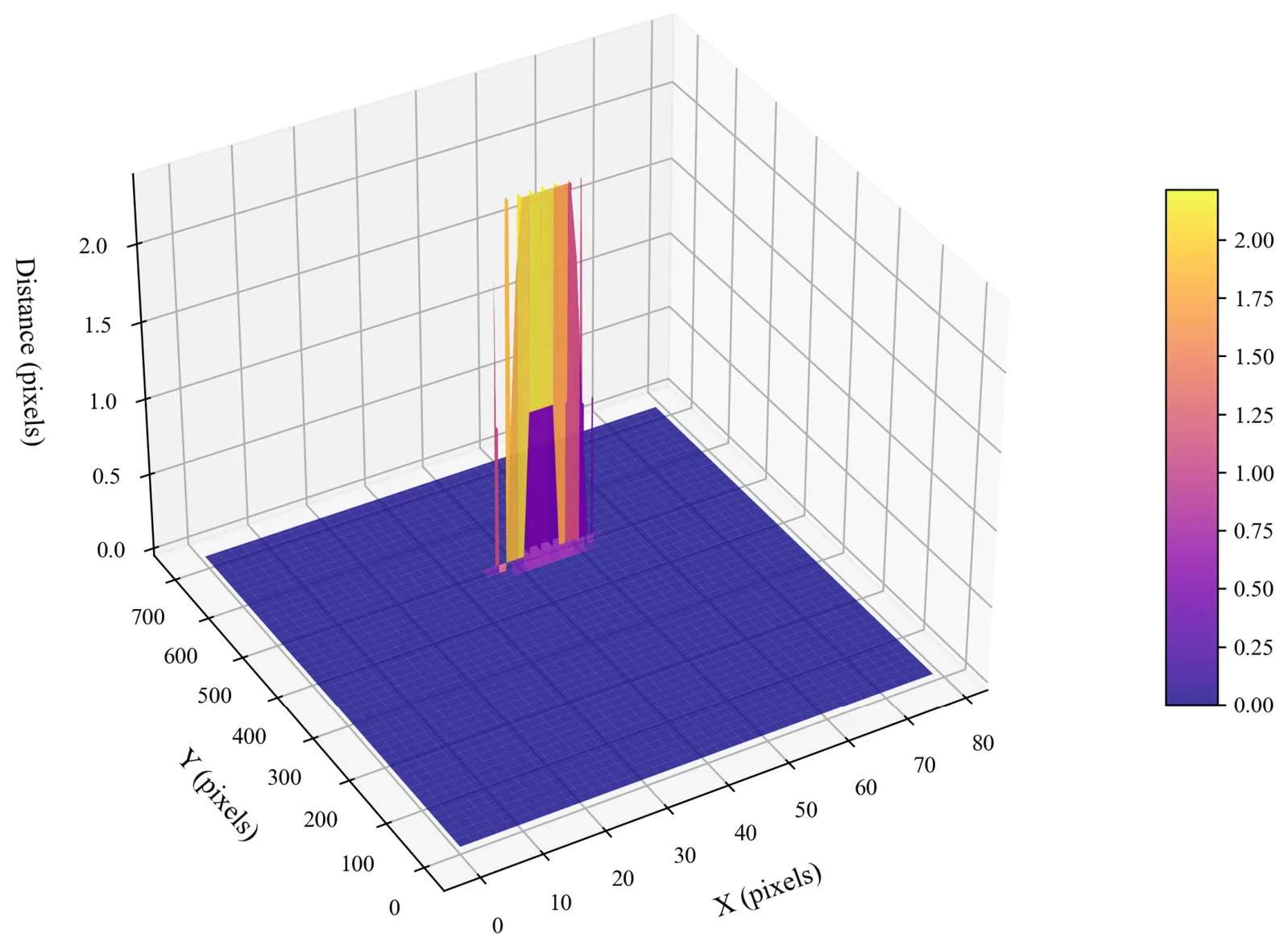
*D*_f_(*x*, *y*) three-dimensional topological graph.

Due to noise interference and slight light intensity fluctuations during image acquisition, *D*(*x*, *y*) contains a large number of false peaks, and direct application of the watershed algorithm will lead to over-segmentation. To eliminate such interference, this paper introduces the H-maxima transform to perform non-maximum suppression on *D*(*x*, *y*). Let the depth filtering threshold *h* be 5, and perform morphological reconstruction on the distance image to flatten the isolated noise peaks with a height less than *h*. The reconstructed filtered distance image *D*_f_(*x*, *y*) is shown in Figure 13:

(11) $$D_{f}(x,y)=\rho_{D}(D-h)$$

where *ρ_D_* represents the morphological geodesic dilation reconstruction of *D* − *h* using *D* as the mask image. The three-dimensional topological map of *D*_f_(*x*, *y*) is shown in Figure 14.

Subsequently, the local maxima of the smoothed surface *D*_f_(*x*, *y*) are extracted as the final internal foreground markers *M*_seed_(*x*, *y*) of potential defect cores:

(12) $$M_{\mathrm{seed}}(x,y)=\left\{\begin{array}{ll} 1, & \mathrm{if}\;D_{f}(x,y)=\underset{k\to \infty }{\lim}\delta_{A}^{k}\left[D_{f}(x,y)\right]\\ 0, & \mathrm{otherwise} \end{array}\right.$$

wherein $\delta_{A}^{k}$ represents the *k*-th classical morphological dilation operation using the 8-neighborhood structural element *A*_8_. Through this mechanism, only one or a group of geometric cores with physical statistical significance will be retained inside each independent defect, effectively suppressing high-frequency noise interference.

In classical marker-controlled watershed, both internal and external background markers are usually required. In our constrained binary setting, however, the defects of interest are the only foreground objects inside the dynamic ROI mask. Therefore, background markers are implicitly provided by the surrounding normal weld pixels and the outer boundary of the dynamic mask *M*_roi_(*x*, *y*). We do not perform explicit background marker generation, marker merging, or forced minima imposition. To convert the peaks of the internal markers into basins suitable for watershed evolution, this paper performs grayscale inversion on the distance transform map *D*(*x*, *y*) to construct an inverted topographic map *D*_inv_(*x*, *y*), converting the defect core regions into valleys with the lowest potential energy, while the narrow bottlenecks at the adhesion points are transformed into higher “saddles”.

Since the standard watershed algorithm tends to generate segmentation lines at the local minima of the distance transform map, in order to convert the peaks in the defect core foreground markers *M*_seed_(*x*, *y*) into basins that can be used for the evolution of the watershed algorithm, it is necessary to perform a grayscale inversion operation on the distance transform map *D*(*x*, *y*) to construct the inverted topographic map *D*_inv_(*x*, *y*):

(13) $$D_{\mathrm{inv}}(x,y)=\underset{\left(u,v\right)\in \Omega_{f}}{\min}D(u,v)-D(x,y)$$

Through inversion, the center of the original defect connected domain is transformed into a basin with the lowest potential energy, while the defect adhesion area is transformed into a saddle with higher potential energy.

**Figure 15:**
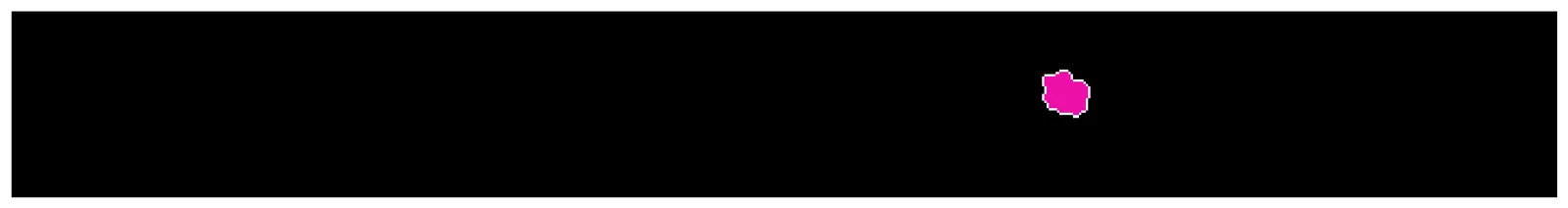
Mark the controlled watershed segmentation results.

Subsequently, with *M*_seed_(*x*, *y*) as the only seed source, the marker-controlled watershed algorithm is used to simulate the morphological evolution of water level rise on the inverted topographic map *D*_inv_(*x*, *y*). Water flows spread upward simultaneously from each independent valley bottom marker. When water flows from different cores meet at the adhered saddle, the algorithm constructs a watershed line there, thereby effectively cutting off the adhered pseudocontour. Finally, a set of *M* completely independent candidate defect connected domains **C** = {*C*_1_, *C*_2_, …, *C_M_*} is output, ensuring the geometric integrity of subsequent feature measurement. The segmentation effect is shown in Figure 15.

#### 3.4.2. Local Background Envelope Construction and Gray Reverse Mapping

To recover the local grayscale information of each candidate defect region after binary segmentation, the spatial coordinates of each independent candidate connected region *C_i_* obtained by the marker-controlled watershed algorithm are mapped back to the grayscale image *I*(*x*, *y*). The mean gray value of the candidate region is calculated as

(14) $$\mu_{\mathrm{defect}}^{i}=\frac{1}{\left|C_{i}\right|}\sum_{(x,y)\in C_{i}}I(x,y)$$

wherein |*C_i_*| represents the total number of pixels contained in the connected domain *C_i_*.

Because the absolute gray level of a candidate region is affected by the local background and imaging conditions, its gray level alone is insufficient to determine the polarity of the defect. Therefore, a local reference region is constructed around each candidate region. Specifically, a 3 × 3 square structuring element *S* is used to dilate *C_i_*, and the candidate region itself is excluded from the dilated region. The local reference region mask is therefore defined as

(15) $$M_{\mathrm{bg}}^{i}=(C_{i}⊕S)-C_{i}$$

wherein ⊕ denotes the morphological dilation operator. Thus, $M_{\mathrm{bg}}^{i}$ represents the immediate surrounding pixels of *C_i_* and provides a local estimate of the normal weld background under nearly identical imaging conditions.

The mean gray value of this local reference region is calculated as

(16) $$\mu_{\mathrm{bg}}^{i}=\frac{1}{\left|M_{\mathrm{bg}}^{i}\right|}\sum_{(x,y)\in M_{\mathrm{bg}}^{i}}I(x,y)$$

where |$M_{\mathrm{bg}}^{i}$| denotes the number of pixels in the local reference region.

This local reference construction is preferable to using a global background intensity because the weld image is affected by spatially non-uniform illumination and the optical scattering characteristics of translucent polypropylene. By comparing each candidate region with its immediate surrounding region, the feature characterizes the local gray-level polarity of the candidate rather than its absolute brightness.

#### 3.4.3. Multi-Criteria Defect Polarity Classification and Pseudo-Defect Filtering

Based on the candidate region mean $\mu_{\mathrm{defect}}^{i}$ and the local reference mean $\mu_{\mathrm{bg}}^{i}$, a local polarity contrast *P_i_* is defined as

(17) $$P_{i}=\mu_{\mathrm{defect}}^{i}-\mu_{\mathrm{bg}}^{i}$$

The feature describes both the magnitude and polarity of the local gray-level difference between a candidate region and its surrounding background. A positive value indicates that the candidate region is brighter than its local background, while a negative value indicates that it is darker.

Under the imaging configuration adopted in this study, a ring-shaped white light source is used for front illumination, and a surface light source is used for transmitted backlight imaging. This configuration is designed to enhance the optical contrast of defects in the translucent polypropylene weld region. Under these established imaging conditions, pore defects generally appear as local bright regions because the optical transmission characteristics around the internal pore produce a positive local gray-level contrast. In contrast, burn-through defects are associated with severe localized charring and optical damage, resulting in a local reduction in transmitted intensity and a dark appearance. Therefore, pore and burn-through defects tend to produce positive and negative *P_i_* values, respectively.

Based on this polarity contrast, the candidate regions are classified according to the following criteria:

1. In laser transmission welding, pores arise from trapped air within the material or from non-uniform beam penetration. In optical images, a pore appears as a local bright spot whose internal gray level is significantly higher than that of the surrounding background, satisfying *P_i_* > T_pore_, where T_pore_ is the positive saliency threshold. If a connected component meets this polarity condition and its pixel area corresponds to a physical size larger than 0.5 mm^2^, it is finally identified as a pore defect.
2. Burn-through is a surface defect induced by excessive laser welding energy, which causes severe localized charring and optical damage to the material. This damage leads to an abrupt drop in transmittance, manifesting as a dark spot in the acquired image. The defect is identified when the polarity condition *P_i_* < −T_burn_ is satisfied, where T_burn_ denotes the negative significance threshold. Once this polarity criterion is met, the region is further examined for its spatial extent. If the area of the dark spot exceeds 0.5 mm^2^, the defect is conclusively classified as burn-through.
3. For regions where the pixel value Pi satisfies the condition −T_burn_ ≤ *P_i_* ≤ T_pore_, the extracted response does not exhibit a significant gray-level step relative to the local background. Such variations are attributed to normal illumination fluctuations or interference from the kiln shell texture. The system therefore identifies these regions as false defects and filters them out accordingly.

The thresholds T_pore_ = 2.0 and T_burn_ = 8.0 are determined from the independent calibration dataset and subsequently verified using a separate production batch, as described in Section 4.3. The final 500-sample test set is not involved in threshold determination or adjustment.

#### 3.4.4. Illumination Sensitivity of the Polarity Contrast

The proposed feature is based on the difference between the candidate region intensity and the intensity of its immediate local reference region rather than on an absolute gray-level threshold. Therefore, for an approximately spatially uniform additive illumination change Δ*I*, the two local means can be expressed as

(18) $$\mu_{{\mathrm{defect}}^{\prime }}^{i}=\mu_{\mathrm{defect}}^{i}+\Delta I$$

(19) $$\mu_{{\mathrm{bg}}^{\prime }}^{i}=\mu_{\mathrm{bg}}^{i}+\Delta I$$

Consequently,

(20) $$P_{i}^{\prime }=\mu_{{\mathrm{defect}}^{\prime }}^{i}-\mu_{{\mathrm{bg}}^{\prime }}^{i}=\mu_{\mathrm{defect}}^{i}-\mu_{\mathrm{bg}}^{i}=P_{i}$$

This indicates that, at the feature definition level, an approximately uniform additive change in illumination is largely cancelled by the local reference subtraction. The use of a local reference region therefore provides greater robustness to global intensity fluctuations than a fixed absolute gray-level threshold.

However, the polarity contrast is not completely illumination-invariant. Spatially non-uniform illumination, specular reflection, local saturation, and changes in the optical transmission path may affect the candidate and reference regions differently, thereby changing *P_i_*. Therefore, the proposed polarity classification should be understood as being robust to moderate global intensity shifts under the established imaging configuration, rather than universally invariant to all illumination conditions.

## 4. Experiments and Analysis

### 4.1. Experimental Preparation

The experimental system consists of an industrial camera, a light source, an industrial PC, a Programmable Logic Controller (PLC), and other necessary mechanical components. The development and verification of software algorithms are completed in the Pycharm environment based on Python 3.9 and the OpenCV library. The platform is equipped with an R9-7940HX processor and an RTX4060 GPU, running on the Windows 11 operating system.

**Figure 16:**
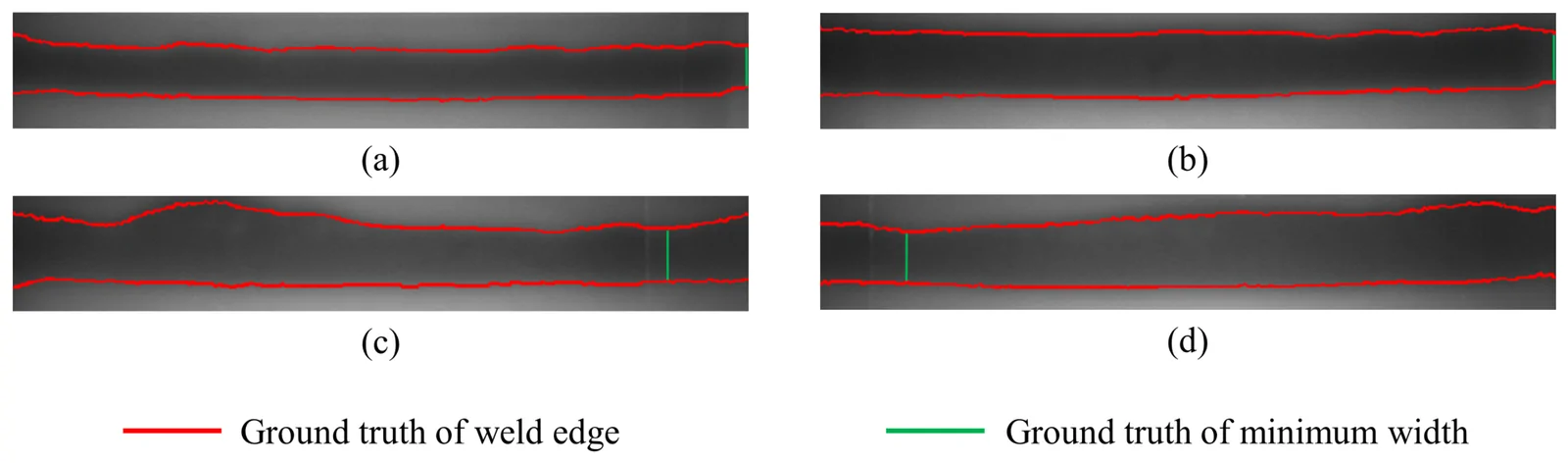
Weld edge and minimum width ground truth annotation. (**a**–**d**) are ground truth annotations of partial data.

To verify the measurement accuracy of the online detection system, this study introduces a 20-megapixel high-resolution industrial camera to establish an offline comparison and verification platform. A dedicated geometric validation dataset comprising 45 weld images of desiccant cartridges is collected for geometric validation. The selected samples cover different weld curvatures, weld widths, and defect states, thereby providing representative variations in weld geometry and appearance for evaluating the proposed weld-boundary extraction and width measurement methods. LabelMe is used to manually annotate the welds along their physical boundaries to obtain the upper and lower weld edges. For each reference point selected on the upper weld edge, the corresponding intersection point on the lower edge is determined strictly along the direction perpendicular to the local mathematical tangent of the upper edge. The distance between the two intersection points is then converted into physical units according to the calibrated pixel-to-millimeter scale and used as the reference width for quantitative evaluation. The annotation results of representative samples are shown in Figure 16.

To verify the repeatability of the ground truth annotation, 15 images were randomly selected from the 45-image dataset and independently re-annotated by a second annotator following the same procedure. The differences between the two annotations were evaluated in terms of weld boundary deviation and calculated weld width error. The average boundary deviation between the two annotators was 0.45 pixels. The corresponding mean absolute difference in the calculated weld width was 0.03 mm The results showed good consistency between the two annotators, supporting the reliability of the manually annotated reference measurements.

To ensure reproducibility, the key parameters used in the image preprocessing stage were fixed throughout all experiments. The corresponding parameter settings are summarized in Table 1.

### 4.2. Accuracy Evaluation of Weld Seam Segmentation Mask

The quality of the weld seam segmentation mask directly determines the reliability of subsequent width measurement and defect extraction. To quantitatively evaluate the performance of the dynamic mask extraction algorithm proposed in this paper, 45 groups of typical desiccant cartridge weld seam images were randomly selected from the dataset for manual annotation experiments. The weld seam contour manually outlined by humans was taken as the ground truth mask, and a pixel-by-pixel comparison was performed with the mask automatically generated by the algorithm.

To quantitatively evaluate the segmentation performance of the algorithm from two dimensions: global region overlap and local boundary fitting accuracy, this paper introduces two comprehensive evaluation metrics: intersection over union (IoU) and mean absolute boundary error (MABE):

(21) $$\mathrm{IoU}=\frac{\left|M_{\mathrm{roi}}\cap M_{\mathrm{gt}}\right|}{\left|M_{\mathrm{roi}}\cup M_{\mathrm{gt}}\right|}$$

where *M*_roi_ represents the dynamic ROI mask set generated by the algorithm in this paper; *M*_gt_ represents the weld ground truth mask set annotated manually.

(22) $$\mathrm{MABE}=\frac{1}{N}\sum_{i=1}^{N}\underset{p_{\mathrm{gt}}\in B_{\mathrm{gt}}}{\min}{\left||p_{\mathrm{alg},i}-p_{\mathrm{gt}}|\right|}_{2}$$

wherein the algorithm-generated boundary point set is *B*_alg_ = {*p*_alg,1_, *p*_alg,2_, …, *p*_alg,*N*_}; the ground truth boundary point set is *B*_gt_; N is the total number of discrete sampling points on the boundary extracted by the algorithm; *p*_alg,*i*_ is the *i*-th pixel coordinate on the algorithm boundary; *p*_gt_ is the search target point on the ground truth boundary; ||·||_2_ represents the Euclidean norm distance in 2D space.

**Figure 17:**
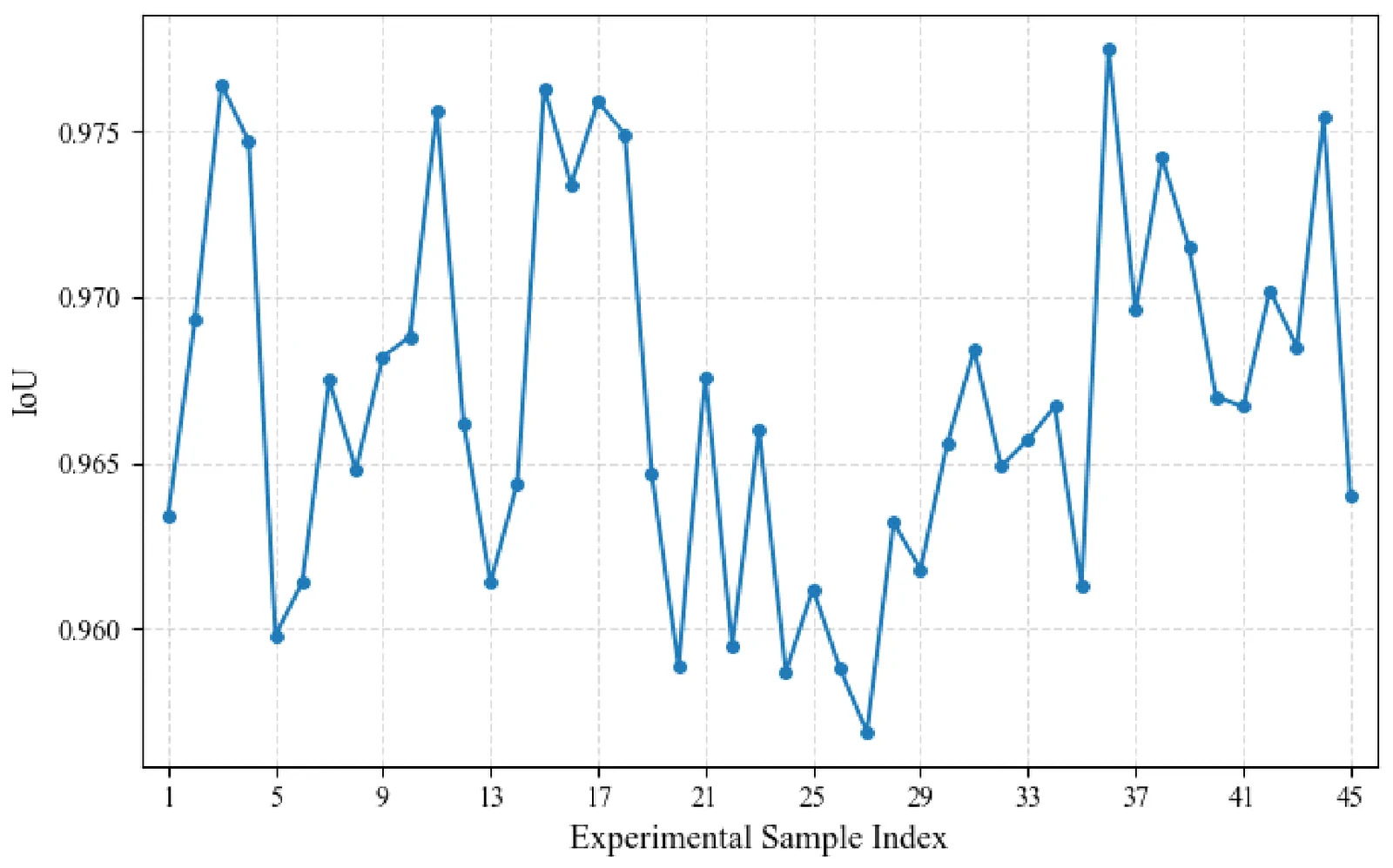
Experimental results of intersection over union.

**Figure 18:**
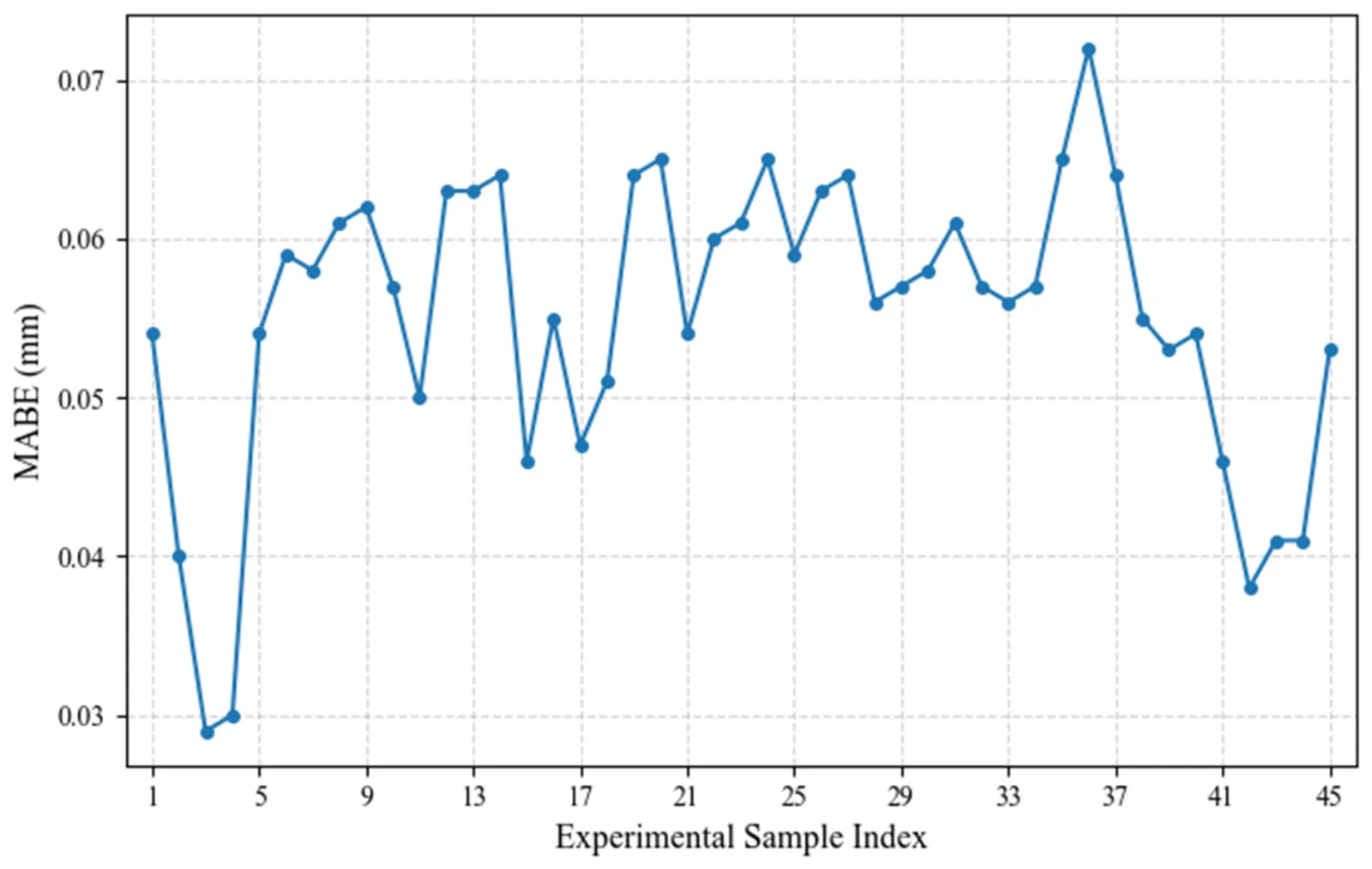
Experimental results of mean absolute boundary error.

**Figure 19:**
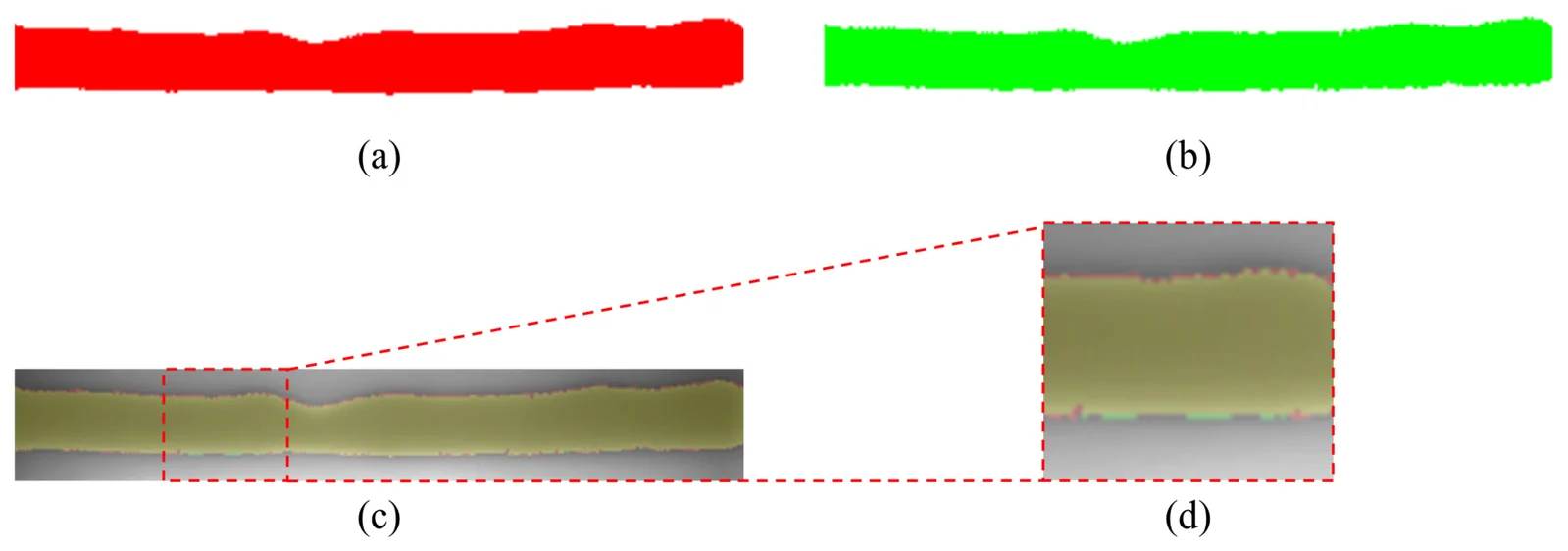
Comparison diagram of the ground truth mask and the generated mask. (**a**) Manually annotated weld ground truth mask. (**b**) Dynamically generated weld mask by the algorithm. (**c**) Overlay of the ground truth mask and the generated mask (the yellow part is the overlapping area). (**d**) Local magnified view.

The Experimental results in Figure 17 and Figure 18 show that the average intersection over union of the algorithm in this paper reaches 96.7%, and the average boundary absolute error is controlled within 0.072 mm. Figure 19 shows a comparison between the ground truth mask and the algorithm-generated mask. The experimental results confirm that the mask generated by the algorithm in this paper has extremely high geometric fidelity, providing an extremely reliable calculation benchmark for subsequent weld width measurement and defect identification.

### 4.3. Discussion on Threshold Selection for Polar Contrast

**Figure 20:**
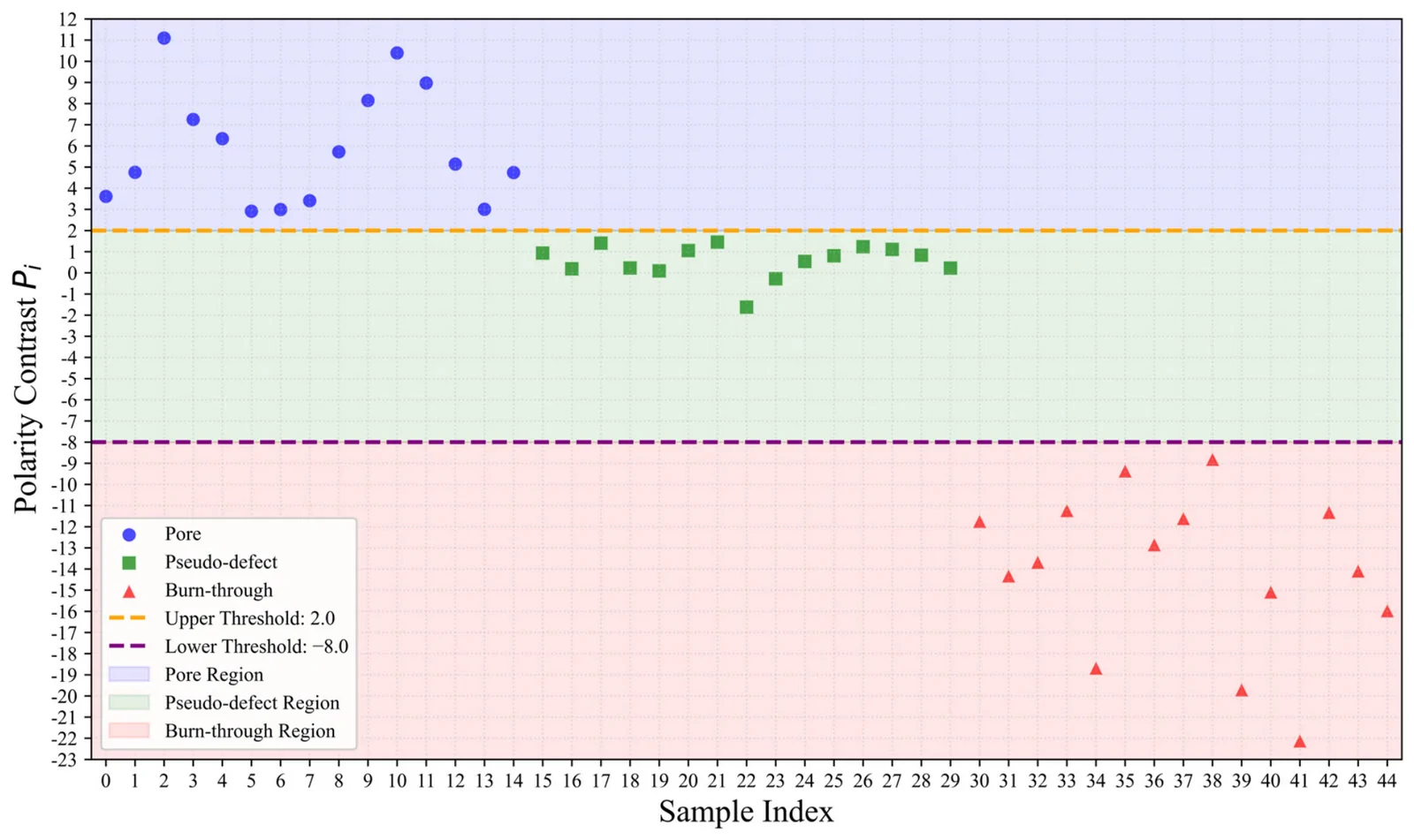
Distribution of polarity contrast *P_i_* for pore defects, burn-through defects, and pseudo-defects in the calibration dataset.

To determine the polarity thresholds for pore and burn-through classification, a dedicated calibration dataset was first used to analyze the distribution of the polarity contrast *P_i_*. The calibration dataset was collected from an independent production batch and contained 45 representative samples, including 15 pore defects, 15 burn-through defects, and 15 pseudo-defect connected domains generated after watershed segmentation. The polarity contrast *P_i_* was calculated for each sample, and the resulting feature distributions were analyzed to determine the positive and negative polarity thresholds. The distribution of the polarity contrast in the calibration dataset is shown in Figure 20. Based on the observed separation between the three categories, the thresholds were set to T_pore_ = 2.0 and T_burn_ = 8.0.

To independently verify the stability of the selected thresholds, a second production batch containing the same three categories of samples was used as a validation dataset. The validation dataset was not involved in determining either threshold. As summarized in Table 2, the polarity contrast of the three categories remained well separated in the validation batch. The minimum polarity value of the pore defects was 2.91, which was higher than T_burn_ = 8.0, whereas the maximum polarity value of the burn-through defects was −8.81, which was lower than T_burn_ = 8.0. The polarity contrast of the pseudo-defects remained within the interval of [−1.87, 1.47], which was located between the two thresholds. These results demonstrate that the selected fixed thresholds remained effective on an independent production batch without further threshold adjustment.

Importantly, the 500 samples used for the final performance evaluation were not involved in either threshold calibration or validation. All parameters were fixed before the evaluation on the 500-sample test set, thereby avoiding the use of test samples for threshold selection and providing an independent assessment of the proposed defect classification method.

It can be intuitively observed from Figure 20 that the three types of regions exhibit significant clustering characteristics in the polarity contrast space. The pseudo-defect regions are mainly caused by uneven illumination and material surface texture, with weak local grayscale fluctuations, and the *P_i_* values are concentrated in a very narrow interval near zero. In contrast, pore and burn-through defects cause strong shifts in polarity values towards the positive and negative poles, respectively, as they alter the physical path of transmitted light. Among them, burn-through defects appear as obvious dark patches in the grayscale image, with a large grayscale difference from the normal weld area, resulting in a high polarity contrast value. Pore defects, on the other hand, appear as relatively indistinct bright spots with a small grayscale difference from the background.

In this paper, the outer envelope line T_pore_ = 2.0 of the pore defect distribution boundary is selected as the pore defect classification threshold to ensure the sensitivity to weak bright spots; the outer envelope line T_burn_ = 8.0 of the burn-through defect distribution boundary is selected as the burn-through defect classification threshold to avoid potential false detection caused by excessive grayscale steps. Through these two polar boundary lines, the system can accurately filter out the illumination fluctuations within the threshold interval [−T_burn_, T_pore_] as pseudo-defects, which confirms the robustness of the grayscale inverse mapping mechanism in defect classification from the data statistics level.

### 4.4. Weld Width Measurement Accuracy Verification

To verify the accuracy and effectiveness of the minimum distance algorithm based on orthogonal projection proposed in this paper, four classical weld width measurement methods widely used in industrial fields, namely the sequence search method [24], the local caliper method, the least squares curve fitting method and the skeleton-based width measurement method were selected for comparison. To ensure a fair comparison, these methods using identical ground truth measurements. The reference weld widths were obtained from manually annotated weld boundaries, and the same annotation and measurement protocol was applied to all methods. Therefore, the comparison results directly reflect the differences in width extraction performance among the five methods. Table 3 presents the error statistical indicators of the three algorithms in terms of average width and minimum width.

**Figure 21:**
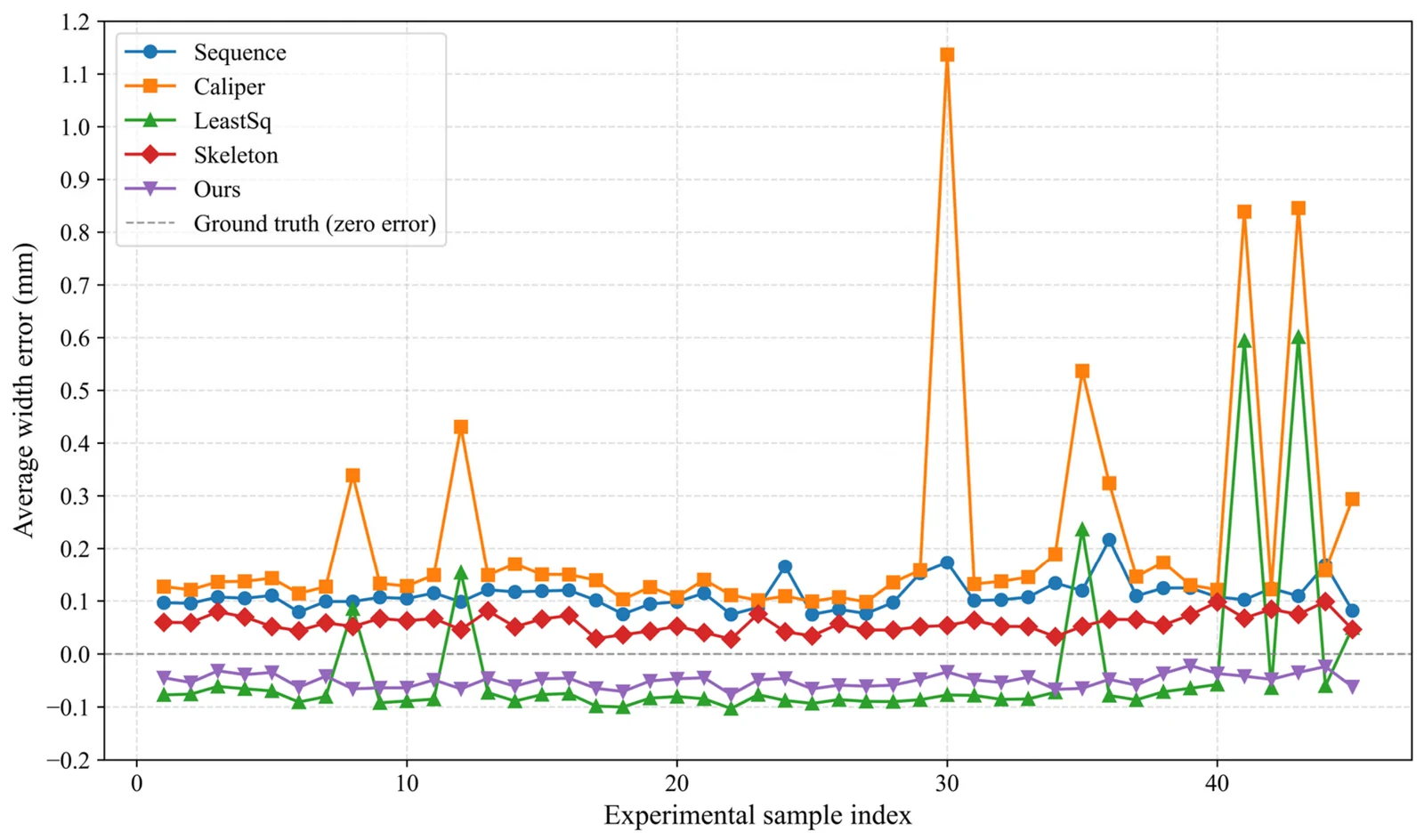
Average width errors of the five measurement methods across 45 experimental samples.

**Figure 22:**
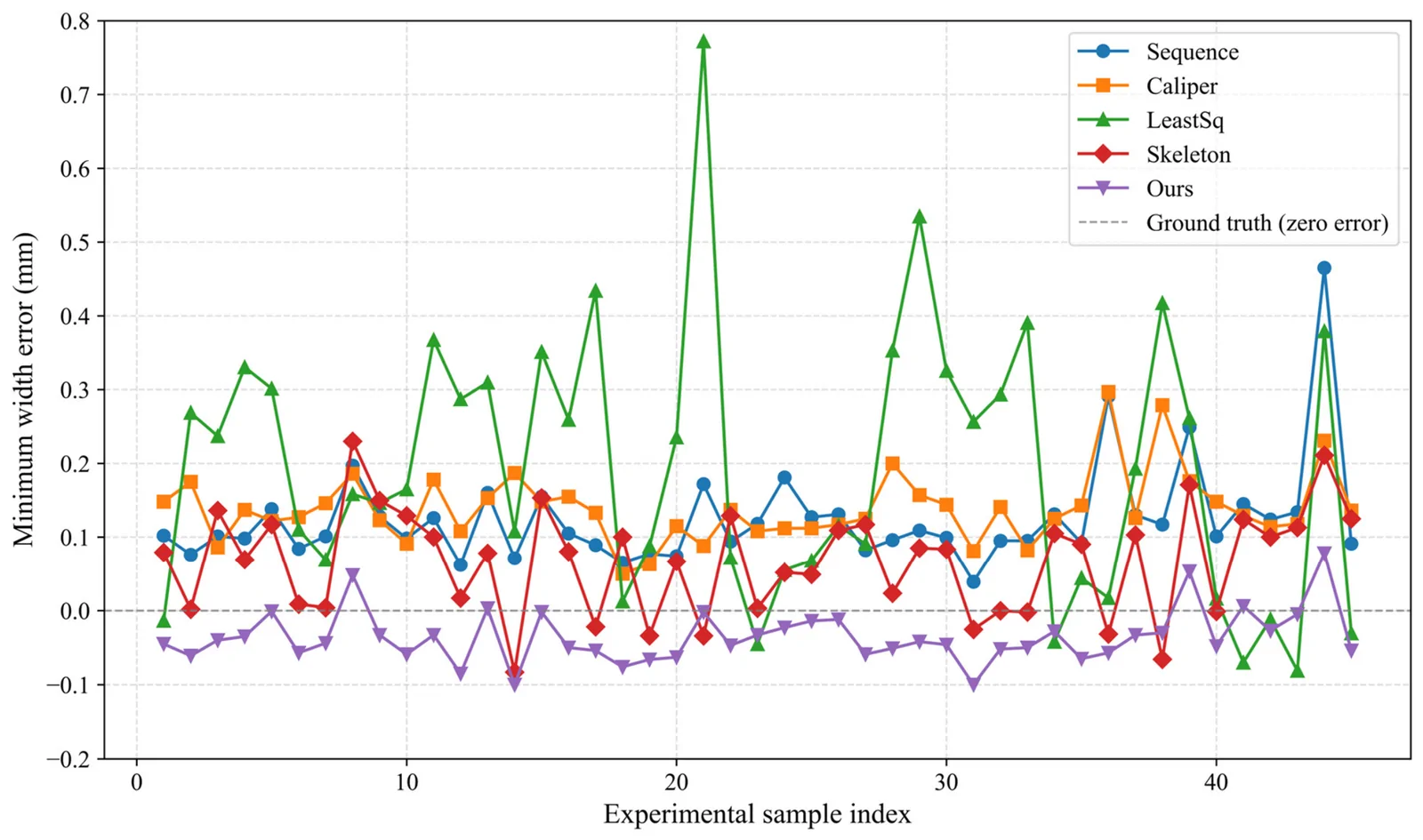
Minimum width errors of the five measurement methods across 45 experimental samples.

To further examine the measurement stability across individual samples, the average width and minimum width errors of all 45 experiments are presented in Figure 21 and Figure 22, respectively. As shown in Figure 21, the proposed method maintains relatively low and stable average width errors across the 45 experiments, with less fluctuation than the other methods. A similar trend can be observed for the minimum width errors in Figure 22, where the proposed method consistently exhibits low errors across different samples. These results further demonstrate the stability of the proposed method in addition to the overall statistical performance reported in Table 3. Comparison of five weld width measurement algorithms.

As can be seen from Table 3, the proposed method achieves the lowest errors among all five methods for both average-width and minimum-width measurements. The proposed method obtains an AW AE of 0.0509 mm, an AW RE of 1.69%, and an AW RMSE of 0.0524 mm. For minimum-width measurement, the corresponding values are 0.0438 mm, 1.72%, and 0.0501 mm, respectively. The skeleton-based method provides the second-best overall accuracy, whereas the least squares curve fitting method shows relatively larger errors in minimum-width measurement. In terms of computational efficiency, the proposed method requires only 1.84 ms per image, which is lower than other methods. These results indicate that the proposed method provides a favorable balance between measurement accuracy and computational efficiency.

**Figure 23:**
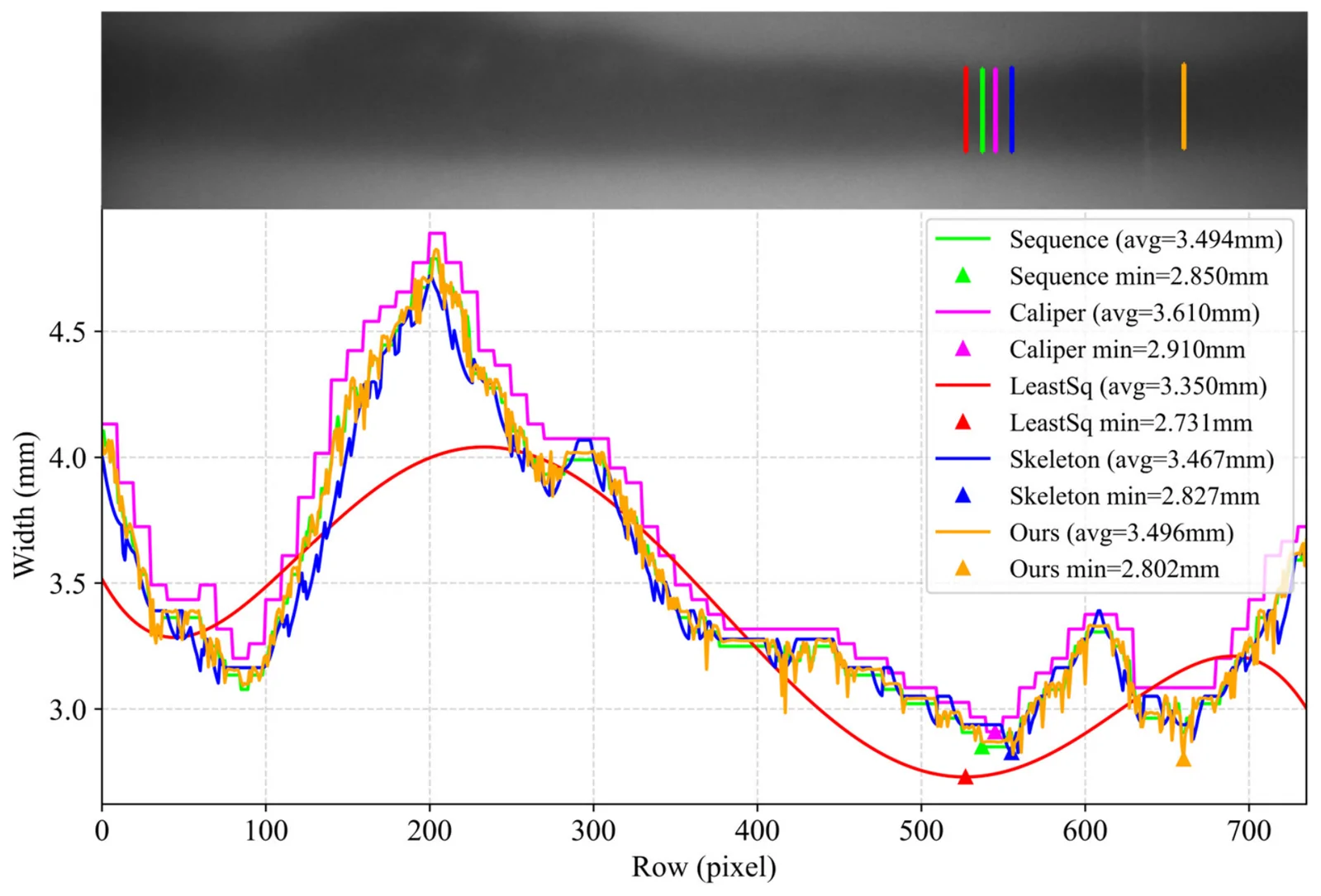
Comparison chart of actual detection effects of five weld width measurement algorithms.

The differences among the methods are further illustrated in Figure 23. The sequence search method is sensitive to the geometric inclination of the weld path because its measurement direction is fixed, while the local caliper method can be affected by local curvature when estimating the minimum width. The least squares curve fitting method provides a global approximation of the two weld boundaries but may introduce errors when the actual weld geometry deviates from the fitted curve. The skeleton-based method adapts to the local weld geometry, but its accuracy depends on the quality and topology of the extracted skeleton. In contrast, the proposed method calculates the minimum Euclidean distance between the fitted weld boundaries along their local normal direction, allowing for it to accommodate curved weld geometries while maintaining low computational cost.

### 4.5. System Comprehensive Performance Evaluation

To comprehensively evaluate the reliability, stability, and execution efficiency of the visual detection algorithm proposed in this paper in actual industrial production environments, a comprehensive performance evaluation is conducted relying on the machine vision experimental platform built in the previous section.

#### 4.5.1. Classification Accuracy Statistics

The test set uses 500 real welding samples of desiccant cartridge randomly collected from industrial sites, including 205 qualified products calibrated by cross-recheck between manual and production line, and 295 defective products with four types of defects. The complete algorithm including image preprocessing, weld segmentation and defect detection and recognition in this paper is applied to the test set. According to the comparison results between the system judgment and the manual real labels, the defect detection performance of the system is evaluated.

**Figure 24:**
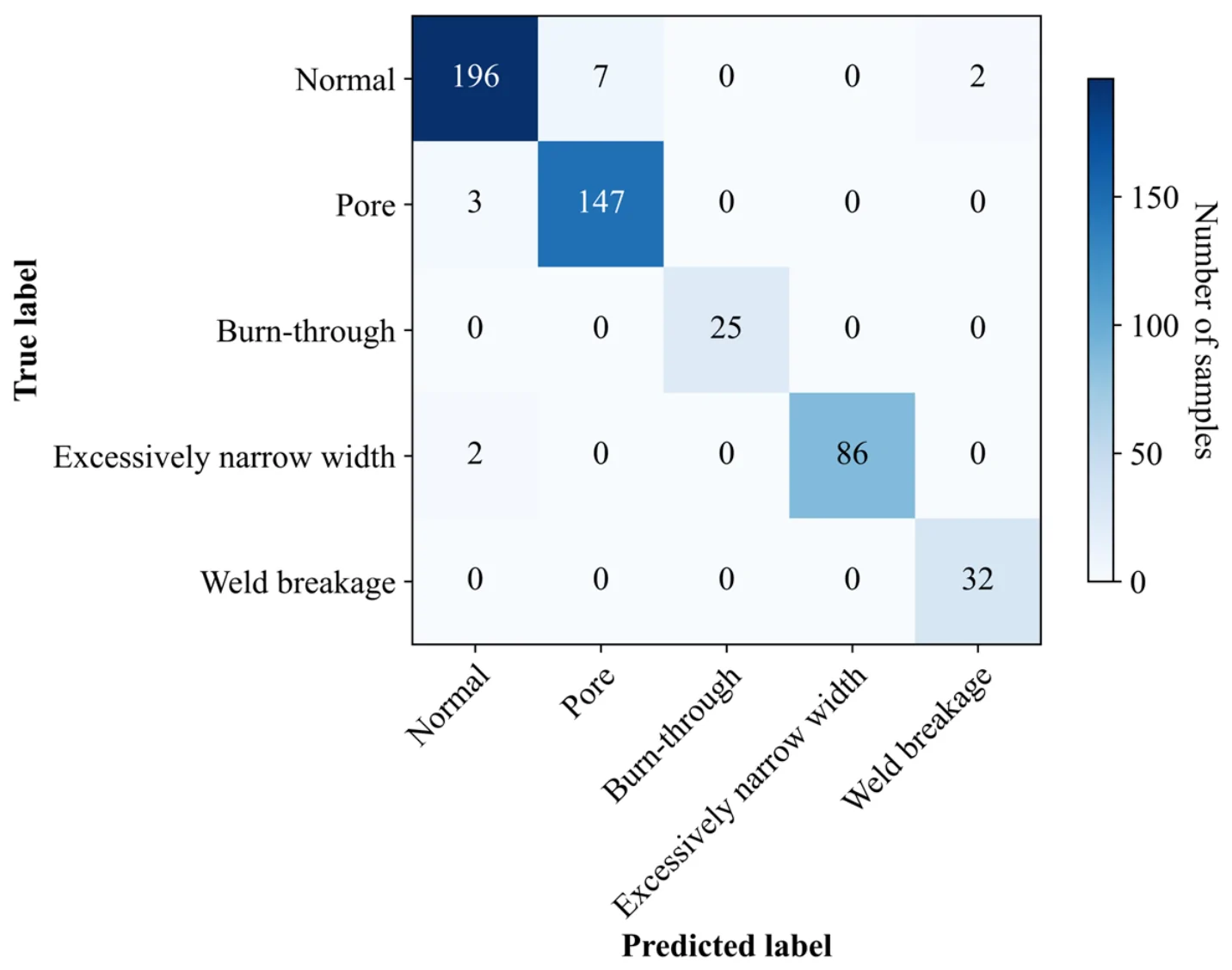
Confusion matrix of the proposed defect detection method on the test set.

The detection results of the proposed algorithm on the 500-sample test set are summarized in Table 4. The test set consists of 205 normal weld samples, 150 pore defect samples, 25 burn-through samples, 88 excessively narrow-width samples, and 32 weld breakage samples. Because of the unequal distribution among defect categories, class-wise Precision, Recall, and F1-score, together with Macro-F1, were used in addition to the overall accuracy to provide a more comprehensive evaluation, the confusion matrix is shown in Figure 24.

**Figure 25:**
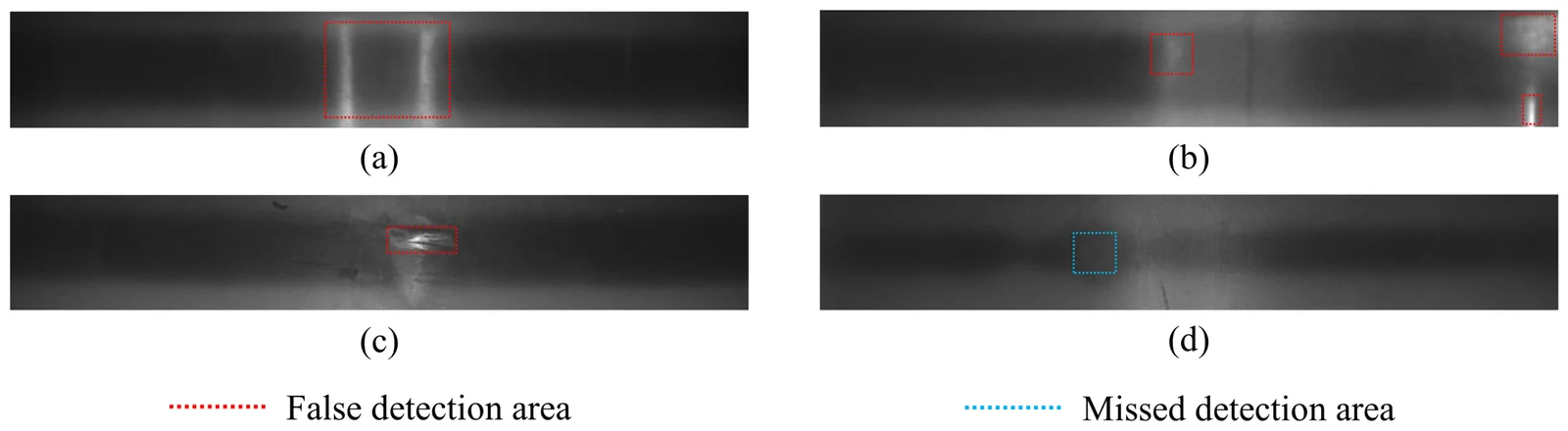
False detection and missed detection diagram: (**a**) Normal weld false detection as weld breakage. (**b**,**c**) Normal weld false detection as pore. (**d**) Pore defect missed detection.

As shown in Table 4, 196 of the 205 normal weld samples were correctly identified, while nine normal samples were falsely classified as defective. Specifically, seven normal samples were classified as pore defects and two were classified as weld breakage, as shown in Figure 25a–c. For the pore defect category, 147 of 150 samples were correctly detected, resulting in a Recall of 98.00%, while the Precision and F1-score were 95.45% and 96.71%, respectively. The three missed pore defects were mainly associated with low contrast between the pores and the surrounding weld region, causing them to be removed as pseudo-defects during subsequent processing, as shown in Figure 25d.

All 25 burn-through samples were correctly identified, yielding a Precision, Recall, and F1-score of 100.00%. Although burn-through is the minority class and accounts for only 5% of the test set, no false positives or false negatives were observed for this category in the present test set.

For excessively narrow width defects, 86 of 88 samples were correctly detected, corresponding to a Recall of 97.73%, a Precision of 100.00%, and an F1-score of 98.85%. The two missed samples were located close to the critical minimum weld width threshold. In these cases, the measured width was slightly larger than the actual width, resulting in their classification as normal welds.

For weld breakage defects, all 32 defective samples were correctly detected. However, two normal samples were falsely classified as weld breakage because strong reflections appeared in the middle region of the cylindrical component at specific viewing angles, producing fracture-like patterns in the weld image. Consequently, the Precision and Recall for weld breakage were 94.12% and 100.00%, respectively, with an F1-score of 96.97%.

Overall, 486 of the 500 test samples were correctly classified, corresponding to an overall accuracy of 97.20%. The Macro-F1 score was 97.82%, indicating that the proposed method maintains a high and relatively balanced performance across the five categories despite the class imbalance in the test set. These additional class-wise metrics provide a more comprehensive assessment than overall accuracy alone. Nevertheless, the remaining false detections and missed detections indicate that strong reflections, low-contrast pores, and weld width measurements close to the critical threshold remain the main sources of detection errors.

**Figure 26:**
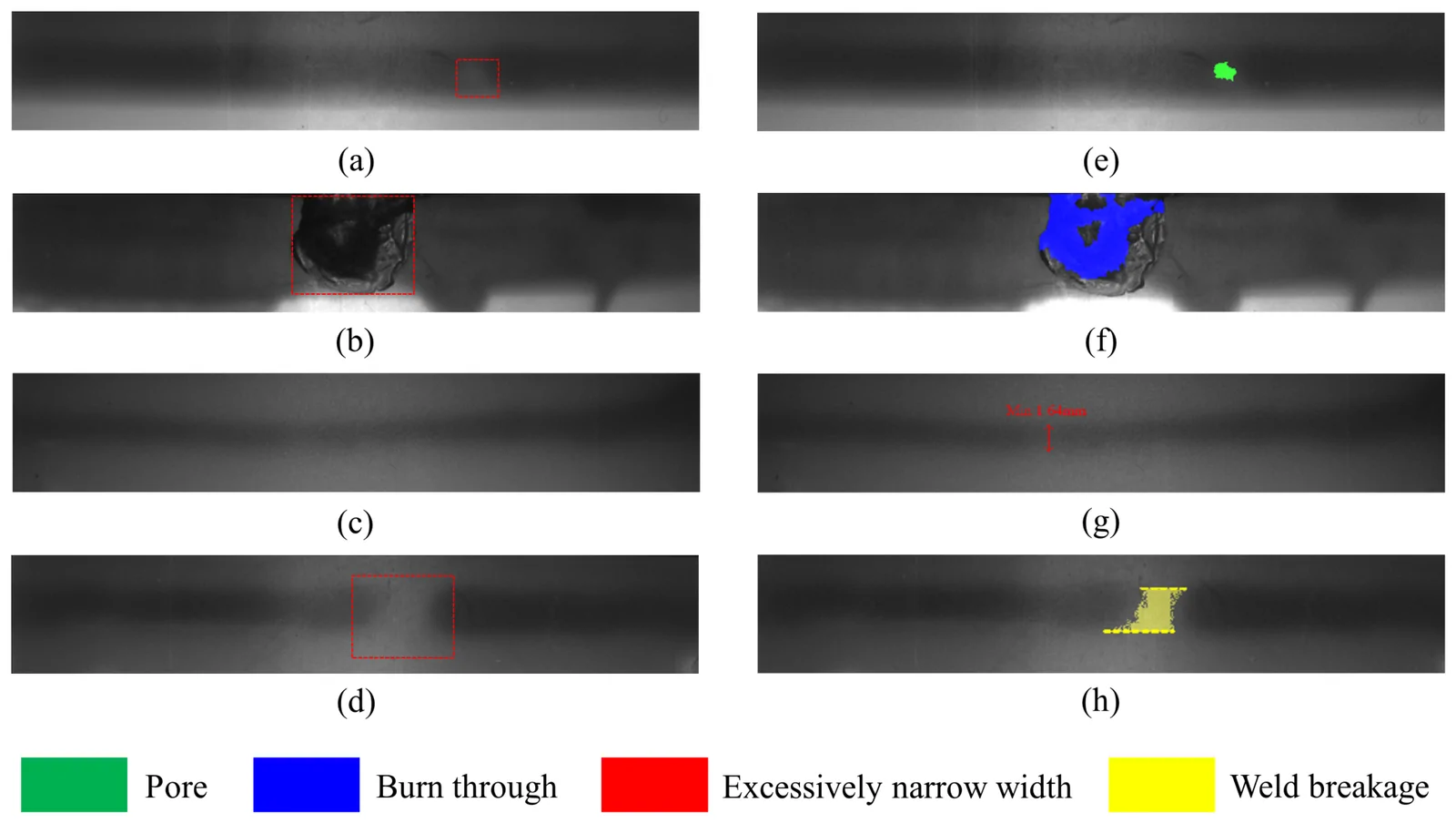
Different types of defects and their detection results: (**a**) pore defect; (**b**) burn-through defect; (**c**) excessively narrow weld defect; (**d**) weld breakage defect; (**e**) detection result of pore defect; (**f**) detection result of burn-through defect; (**g**) detection result of excessively narrow weld defect; (**h**) detection result of weld breakage defect.

Figure 26 shows the detection effects of various types of defects. Overall, the proposed defect detection algorithm achieved an overall accuracy of 97.20% and a Macro-F1 score of 97.82% on the test set, demonstrating its effectiveness for automated weld quality inspection of desiccant cartridges under the evaluated industrial imaging conditions.

To further characterize the practical detection capability of the proposed system, the physical areas of the successfully detected defects in the independent 500-sample test set were examined. The smallest successfully detected pore defect had an area of 0.608 mm^2^, while the smallest successfully detected burn-through defect had an area of 2.023 mm^2^. These values represent the smallest successfully detected defects observed in the tested production samples rather than absolute detection limits. The 0.5 mm^2^ value used in the algorithm is a candidate region area threshold for filtering small segmented regions and should not be interpreted as an experimentally established minimum detectable defect size. Moreover, the spatial resolution of approximately 0.06 mm/pixel represents the image sampling interval and does not directly determine the minimum detectable physical area. The practical detection capability is jointly affected by defect size and morphology, image contrast, segmentation accuracy, polarity classification, and the candidate region area criterion.

#### 4.5.2. Real-Time Analysis of Algorithms

To evaluate the time complexity of the algorithm and hardware execution efficiency, continuous batch detection is performed on the samples in the test set. The specific time-consuming statistics of each stage are shown in Table 5.

As shown in Table 5, the average end-to-end total processing time of the system is only 55.48 ms. Among them, the extraction of the cubic B-spline dynamic mask in the weld segmentation module involves two traversals of the weld area, accounting for the largest proportion of time consumption, while the image preprocessing and defect recognition modules show high execution efficiency. It fully verifies that the proposed vision inspection algorithm achieves real-time performance and satisfies the computational requirement of the production line inspection process.

## 5. Conclusions

This paper proposes an effective method for weld width measurement and defects detection in laser transmission welding of desiccant cartridge. The core of this method lies in utilizing the geometric continuous boundary features and grayscale polarity information of the weld seam of desiccant cartridge to achieve the extraction and classification of four typical defects. First, dynamic weld mask extraction is used to suppress the interference of non-weld areas; then, based on the geometric continuous boundary features of the weld seam, the orthogonal projection method is used to calculate the weld width to detect excessively narrow weld and weld breakage defects; finally, according to the grayscale polarity information of the weld seam, blowhole and burn-through defects are extracted and classified. Experimental results showed that the proposed method achieved an IoU of 96.7% for weld region extraction. The average-width and minimum-width absolute errors were 0.0509 mm and 0.0438 mm, respectively, with relative errors of 1.69% and 1.72%. Compared with four representative geometric measurement methods, the proposed method achieved the lowest measurement errors and required only 1.84 ms for width measurement. On the independent 500-sample test set, the system achieved an overall defect detection accuracy of 97.20% and a Macro-F1 score of 97.82%, with an average end-to-end processing time of 55.48 ms per sample, which meets the requirements of online inspection under the controlled imaging and production conditions of the target production line.

However, this study still has certain limitations. First, the current method is mainly designed for desiccant cartridge of specific specifications and shapes, and its generalization ability for complex and variable shapes or welding structures of different materials needs to be further verified. Secondly, although this study ensures real-time performance and accuracy through traditional image processing algorithms, the anti-interference robustness of the algorithm still has room for improvement when facing more complex ambient light disturbances and severe surface contamination in industrial sites. Therefore, our future research will focus on collecting more diverse on-site production data to verify the applicability of this method in different product models and new welding technologies. At the same time, we plan to combine the existing geometric feature extraction methods with deep learning technologies to further improve the robustness of the algorithm in extremely harsh industrial environments and the intelligent recognition ability of multi-target defects. In addition, we have identified the detection of further defect types, including cracks, inclusions, and weld displacement, as a key future direction, which may be enabled by integrating higher-resolution imaging, multi-modal sensing, or 3D geometric information.

## Figures and Tables

**Table 1:** Key parameters of the image preprocessing procedure.

Processing Step	Parameter	Value
Median filtering	Kernel size	10 × 20
Laplacian sharpening	Sharpening strength	135
	Kernel size	51 × 51
Histogram equalization	Contrast coefficient	140
Sauvola binarization	Correction coefficient *k*	0.15
	Dynamic range *R*	60
	Kernel size	40 × 40
Morphological processing	Operation	Opening followed by closing
	Kernel size	3 × 3

**Table 2:** Polarity contrast ranges across two production batches.

Dataset	Category	Min *P_i_*	Max *P_i_*	Threshold Criterion
Calibration	Pore	2.91	11.10	*P_i_* > 2.0
	Burn-through	−22.12	−8.81	*P_i_* < −8.0
	Pseudo-defect	−1.62	−1.45	−8.0 ≤ *P_i_* ≤ 2.0
Validation	Pore	2.70	11.89	*P_i_* > 2.0
	Burn-through	−20.84	−8.96	*P_i_* < −8.0
	Pseudo-defect	−0.13	1.47	−8.0 ≤ *P_i_* ≤ 2.0

**Table 3:** Comparison of five weld width measurement algorithms.

Methods	Average Width				Minimum Width				Time/ms
	AE * (mm)	RE *	RMSE (mm)	SD (mm)	AE * (mm)	RE *	RMSE (mm)	SD (mm)	
Sequence	0.111	3.654%	0.114	0.0281	0.125	4.97%	0.142	0.0696	27.50
Caliper	0.215	7.13%	0.304	0.218	0.139	5.49%	0.147	0.0480	3.14
LS	0.108	3.651%	0.154	0.110	0.204	8.49%	0.262	0.165	3.26
Skeleton	0.0576	1.93%	0.0599	0.0165	0.0802	3.19%	0.0979	0.0722	55.91
Ours	0.0509	1.69%	0.0524	0.0127	0.0438	1.72%	0.0501	0.0246	1.84

* AE: mean absolute error; RE: mean relative error.

**Table 4:** Defect detection results.

Class	Sample Size	Proportion	TP	FP	FN	Precision	Recall	F1-Score
Normal	205	41%	196	5	9	97.51%	95.61%	96.55%
Pore	150	30%	147	7	3	95.45%	98.00%	96.71%
Burn-through	25	5%	25	0	0	100.00%	100.00%	100.00%
Excessively narrow width	88	18%	86	0	2	100.00%	97.73%	98.85%
Weld breakage	32	6%	32	2	0	94.12%	100.00%	96.97%
Overall accuracy	500	100%	486	—	—	—	—	97.20%
Macro-F1	—	—	—	—	—	—	—	97.82%

**Table 5:** Statistics on the average time consumption of each module in visual detection algorithms.

Algorithm Module	Core Execution Steps	Time/ms	Proportion of Total
Image Preprocessing	Median filtering	2.75	9%
	Laplacian sharpening	1.30	
	Histogram equalization	0.99	
Weld seam segmentation	Sauvola binarization	2.46	74%
	Morphological processing	2.39	
	Dynamic mask extraction	36.36	
Defect identification	Weld seam width measurement	1.85	17%
	Defect connected component extraction	4.04	
	Grayscale polarity discrimination	3.34	
Total system time	End-to-end detection process	55.48	100%

## Data Availability

Data are not publicly available and can be obtained by contacting the corresponding author if necessary.
